# Metal-Mediated Catalytic Polarization Transfer from *para* Hydrogen to 3,5-Dihalogenated Pyridines

**DOI:** 10.1021/acscatal.3c05378

**Published:** 2024-01-05

**Authors:** Ben. J. Tickner, Marcus Dennington, Benjamin G. Collins, Callum A. Gater, Theo F. N. Tanner, Adrian C. Whitwood, Peter J. Rayner, Daniel P. Watts, Simon B. Duckett

**Affiliations:** † Centre for Hyperpolarisation in Magnetic Resonance, University of York, Heslington YO10 5NY, U.K.; ‡ Department of Chemistry, University of York, Heslington YO10 5DD, U.K.; § Department of Physics, Engineering and Technology, University of York, Heslington YO10 5DD, U.K.

**Keywords:** SABRE catalysis, iridium, NMR, hyperpolarization, pyridine, parahydrogen

## Abstract

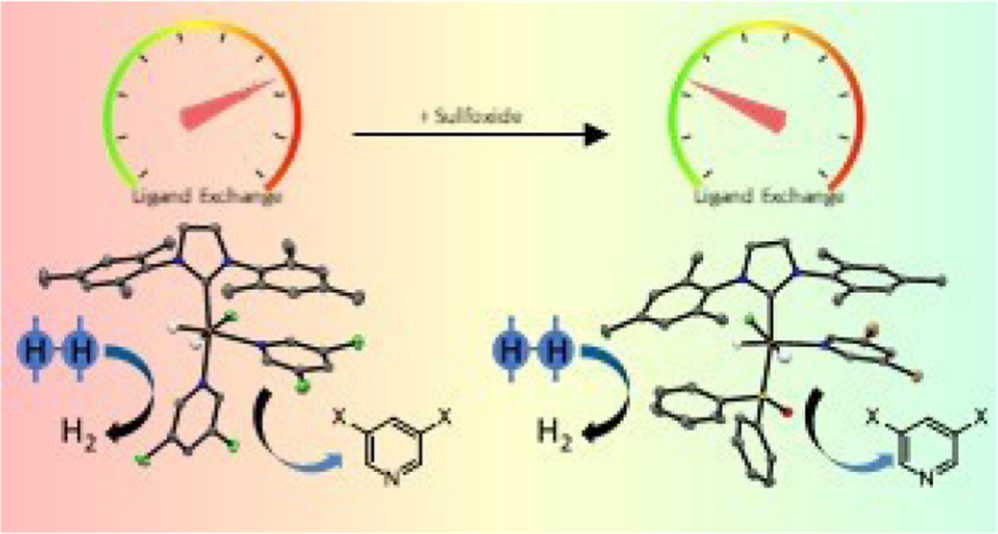

The neutral catalysts [IrCl(H)_2_(NHC)(substrate)_2_] or [IrCl(H)_2_(NHC)(substrate)(sulfoxide)] are used to transfer polarization from *para* hydrogen (*p*H_2_) to 3,5-dichloropyridine and 3,5-dibromopyridine substrates. This is achieved in a rapid, reversible, and low-cost process that relies on ligand exchange within the active catalyst. Notably, the sulfoxide-containing catalyst systems produced NMR signal enhancements between 1 and 2 orders of magnitude larger than its unmodified counterpart. Consequently, this signal amplification by reversible exchange hyperpolarization method can boost the ^1^H, ^13^C, and ^15^N nuclear magnetic resonance (NMR) signal intensities by factors up to 4350, 1550, and 46,600, respectively (14.0, 1.3, and 15.4% polarization). In this paper, NMR and X-ray crystallography are used to map the evolution of catalytically important species and provide mechanistic rational for catalytic efficiency. Furthermore, applications in spontaneous radiofrequency amplification by stimulated emission and NMR reaction monitoring are also shown.

## Introduction

The emerging hyperpolarization technique signal amplification by reversible exchange (SABRE) uses a metal catalyst to transfer polarization from *para* hydrogen (*p*H_2_) to a target molecule.^1,2^ In SABRE, *para* hydrogen (*p*H_2_) is used as the polarization feedstock, which is simply a spin isomer of dihydrogen that is cheap and straightforward to produce.^3^*p*H_2_ itself is NMR-silent, but SABRE unlocks its latent magnetism through a magnetic symmetry breaking reaction. This results from its oxidative addition to a metal center, usually iridium, which generates a metal dihydride complex whose hydride ligand NMR signals can be dramatically sensitized to NMR detection.^4^^1^H magnetization from these enhanced hydride ligands can transfer to other spin-active nuclei in this complex via the *J*-coupling network.^2,5−7^ Consequently, polarization transfer is spontaneous at mT fields for ^1^H nuclei, or at μT fields for heteronuclei.^8,9^ SABRE enables hyperpolarization of free ligands providing there is reversible binding of both *p*H_2_ and the ligand of interest.^10,11^ Therefore, the metal SABRE complex acts to catalyze the transfer of spin order from *p*H_2_ to a free ligand without a chemical change. This is a huge advantage as it produces non-Boltzmann population differences within nuclear spin energy levels which can lead to substantially larger NMR signal intensities than would typically be recorded.^12,13^ This change enables the NMR detection of molecules with short lifetimes or low intrinsic concentrations, which is often challenging using standard NMR methods due to its low sensitivity.

**Figure 1:**
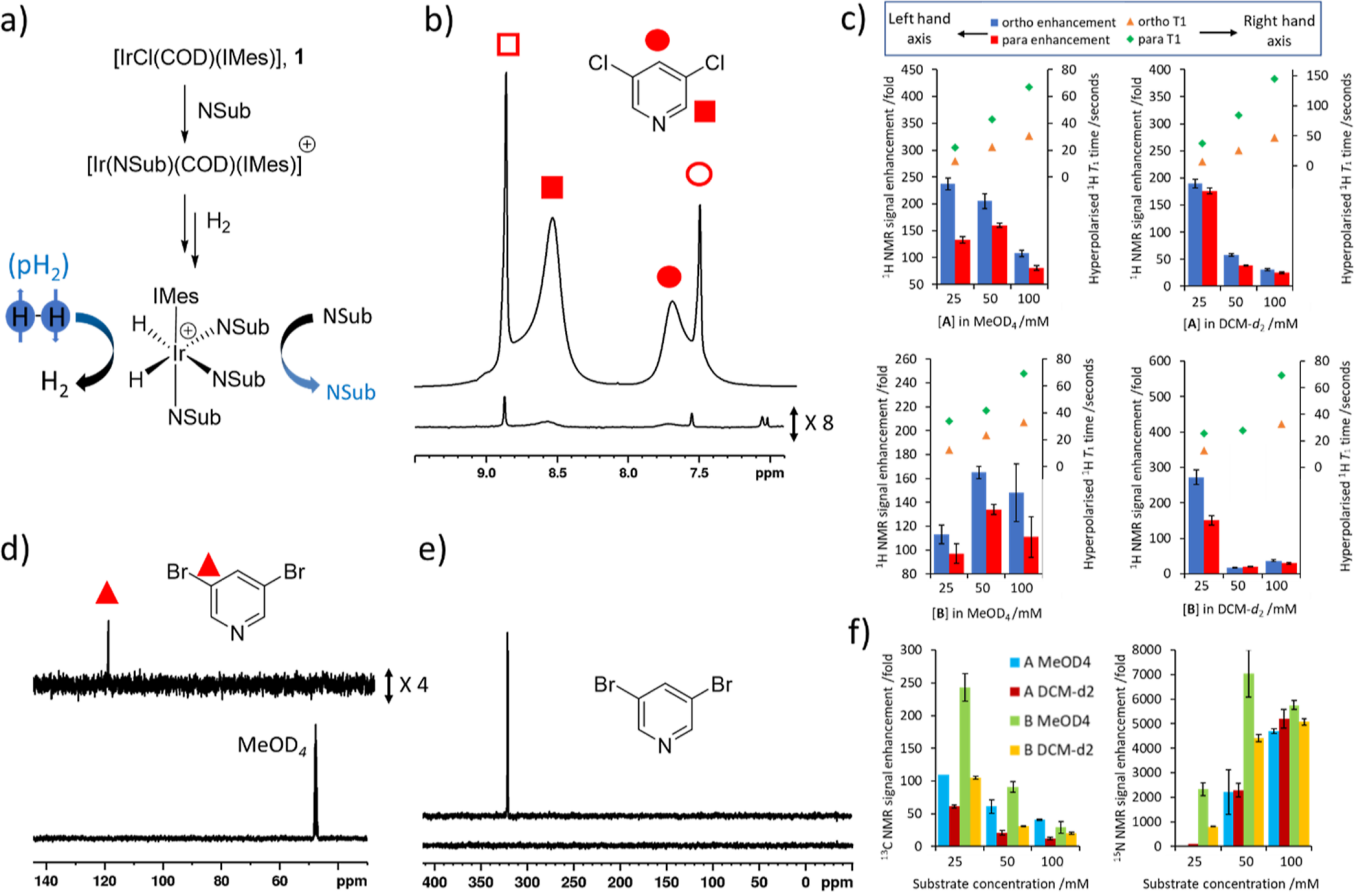
(a) Depiction of the SABRE hyperpolarization process whereby an iridium precatalyst, **1**, is reacted with a substrate and H_2_ to form a typical SABRE catalyst of the form [Ir(H)_2_(IMes)(NSub)_3_]Cl where NSub is an N-donor substrate. The SABRE effect is observed when the complex undergoes reversible exchange of both parahydrogen and substrate. In this work, NSub is 3,5-dichloropyridine (**A**) or 3,5-dibromopyridine (**B**). (b) Representative SABRE hyperpolarized ^1^H NMR spectrum when **1** (5 mM) and **A** (25 mM) are shaken with 3 bar *p*H_2_ for 10 s at *ca* 6.5 mT in methanol-*d*_4_. Resonances marked with filled shapes refer to sites in free **A**, whereas outline shapes indicate the analogous site bound in [IrCl(H)_2_(IMes)(**A**)_2_]. Resonances for free **A** are broadened due to exchange with the metal center, and the effect is more pronounced when lower ligand excesses relative to Ir are used. (c) The effect of substrate loading and solvent on ^1^H NMR signals enhancements (left-hand axis) and ^1^H *T*_1_ times (right-hand axis) for **A** in methanol-*d*_4_ (upper left) and dichloromethane-*d*_2_ (upper right) and **B** in methanol-*d*_4_ (lower left) and dichloromethane-*d*_2_ (lower right). (d) SABRE hyperpolarized ^13^C NMR spectrum when **1** (5 mM) and **B** (25 mM) in dichloromethane-*d*_2_ are shaken with 3 bar *p*H_2_ for 10 s at 1 μT. (e) SABRE hyperpolarized ^15^N NMR spectrum when **1** (5 mM) and **B** (50 mM) in dichloromethane-*d*_2_ are shaken with 3 bar *p*H_2_ for 10 s at 6 μT. (f) The effect of substrate loading and solvent on *meta*^13^C (left) and ^15^N (right) NMR signals enhancements. Note that in (b), (d) and (e) thermally polarized spectra are shown below the hyperpolarized counterpart.

SABRE typically exploits charged polarization transfer catalysts of the type [Ir(H)_2_(IMes)(substrate)_3_]Cl (where IMes is 1,3-bis(2,4,6-trimethyl-phenyl)imidazole-2-ylidene), which form by reaction of [Ir(IMes)(η^2^-η^**2**^-COD)Cl] (where COD is *cis*,*cis*-1,5-cyclooctadiene) with the selected substrate and *p*H_2_ (Figure 1a).^13^ Complexes of this type have proven to enhance the NMR signals of many types of N-donor ligand that ligate to iridium.^2,14−19^ Subtle variations of these catalysts, by adding a coligand, forms species of the type [Ir(H)_2_(IMes)(coligand)(substrate)_2_]Cl or [Ir(H)_2_(IMes)(coligand)(η^2^-substrate)]Cl, which allows sterically hindered, or weakly ligating substrates, to become amenable to SABRE.^20−23^

In this work, we investigate the hyperpolarization of the electron-poor substrates, 3,5-dichloropyridine (**A**) and 3,5-dibromopyridine (**B**), which might be expected to form [Ir(H)_2_(IMes)(substrate)_3_]Cl after reaction with [IrCl(COD)(IMes)] (**1**) and H_2_. In fact, the unusual neutral products [IrCl(H)_2_(IMes)(substrate)_2_] are detected. We measure the resulting ^1^H, ^13^C and ^15^N NMR signal enhancements using SABRE and monitor the reaction leading to these products. Novel iridium species and intermediates are detected, identified, and in some cases analyzed by X-ray diffraction. These studies are then extended to show that significantly higher polarization levels can be attained when one of their dihalopyridine ligands is replaced by a sulfoxide ligand. The resulting [IrCl(H)_2_(NHC)(substrate)(sulfoxide)] complexes are investigated by X-ray crystallography and ligand exchange studies, and the results compared to those obtained for [IrCl(H)_2_(NHC)(substrate)_2_] to rationalize this improvement. This study finishes by demonstrating that these dihalogenated pyridine substrates can be used in several hyperpolarized NMR experiments, including spontaneous radiofrequency amplification by stimulated emission (RASER)^24−27^ and reaction monitoring (using hyperpolarized ^1^H and ^15^N NMR).^28−32^ Hence, this study adds significantly to our understanding of SABRE and its utilization.

## Results and Discussion

### SABRE Hyperpolarization of 3,5-Dichloropyridine (**A**) and 3,5-Dibromopyridine (**B**)

A series of samples were prepared with **1**, and the substrates 3,5-dichloropyridine (**A**) or 3,5-dibromopyridine (**B**) at 25, 50, and 100 mM concentrations (loadings of 5, 10, or 20 equiv relative to the metal) in both methanol-*d*_4_ and dichloromethane-*d*_2_ (0.6 mL). Each NMR sample was then exposed to H_2_ (3 bar) and when the reaction was complete, the samples were shaken with *p*H_2_ (3 bar) for 10 s in the stray field of a 9.4 T magnet and placed immediately into the spectrometer for single scan NMR detection. Hyperpolarized NMR signals are transient and their intensity decreases to the Boltzmann-dictated level according to *T*_1_ relaxation. Consequently for statistical relevance, the hyperpolarization process was repeated multiple times for each sample by replacing the spent *p*H_2_ with fresh *p*H_2_ before reshaking the solution to regenerate enhanced NMR signals.

When single-scan ^1^H NMR measurements were examined in this way, enhanced resonances were observed for both **A** or **B** in all samples. The *ortho* and *para*^1^H NMR signals of **A** proved to be enhanced by 237 ± 11 and 133 ± 6 times their original intensity respectively at 25 mM in methanol-*d*_4_. While the corresponding enhancement values proved similar at 50 mM, they dropped at 100 mM. The analogous measurements in dichloromethane-*d*_2_ resulted in comparable ^1^H NMR signal enhancements to those in methanol-*d*_4_ at 25 mM, although they were significantly smaller than those in methanol-*d*_4_ at 50 mM and 100 mM. Similar behavior was seen for **B**, with the ^1^H NMR signal enhancements reaching a value of 272 ± 21-fold and 151 ± 13-fold for a 25 mM loading in dichloromethane-*d*_2_ (Figure 1 and the Supporting Information S1 for values).

The longitudinal relaxation time constants (*T*_1_) of hyperpolarized resonances can be a dominant factor in controlling the observed NMR signal enhancements. Accordingly, single-shot hyperpolarized *T*_1_ values were also measured for these samples (Figure 1c). Generally the values for both **A** and **B**, in either solvent, are shortened by the presence of the metal catalyst, and this effect is lessened when the substrate is present at a higher loading.^33−35^ For example, the *T*_1_ times extend to 170 and 262 s for the *ortho* and *para* sites, respectively of **A** when there is a 200-fold excess of it compared to **1** (Table S5). When the catalyst is present at 5 equiv relative to **A**, these *T*_1_ times compress to just 9% and 24% of these values (Table S5). It is therefore clear that for ^1^H hyperpolarization, relaxation is not limiting as these values are much longer than the expected polarization transfer times. Rather, differences in polarization level should relate to ligand exchange rates, which are discussed later. Nevertheless, a key finding of this study is that **A** and **B** contain some of the longest reported hyperpolarized ^1^H *T*_1_ times.^36−40^

SABRE also enhances the ^13^C and ^15^N signals of **A** and **B** when these samples are shaken at magnetic fields of 1 and 6 μT, respectively (Figure 1d,e) to achieve direct polarization transfer from the hydride ligands to heteronuclei.^9,21^ For example, a ^13^C NMR signal for the *meta* resonance of **A** is detected in both methanol-*d*_4_ and dichloromethane-*d*_2_ with enhancements of 110-fold and 61-fold, respectively, which again drop as the substrate loading is increased (Figure 1f and the Supporting Information S1, Table S3). The ^13^C NMR signal enhancement for the *meta*^13^C site of **B** is larger in methanol-*d*_4_ (243 ± 21-fold) than in dichloromethane-*d*_2_ (105 ± 2-fold), and it also decreases as the substrate loading is increased. This enhanced ^13^C NMR signal appears as an in-phase signal, which is indicative of direct polarization transfer from the hydride ligands to ^13^C via SABRE–SHEATH at these 1-μT polarization transfer fields (Figure 1d).

Excitingly, significant ^15^N NMR signal enhancements can be achieved for free **A** or **B**. In contrast to ^1^H and ^13^C hyperpolarization trends, they appear to increase as the substrate loading is increased (Figure 1f and the Supporting Information S1, Table S4) with the highest values of 4702 ± 91-fold and 5203 ± 387-fold achieved for **A** and 7044 ± 961-fold and 5077 ± 131-fold for **B** in methanol-*d*_4_ and dichloromethane-*d*_2_, respectively. Overall, **B** appeared to be more efficiently hyperpolarized under these conditions than **A** which is rationalized in more detail in the ligand exchange section.

These results highlight that the SABRE hyperpolarization of the electron poor **A** and **B** can be achieved, and indicate that a stable polarization transfer catalyst is formed in each case. Indeed, the ^1^H NMR experiments involving **A** or **B** reveal enhanced hydride NMR signals at δ −24.02 and δ −24.66 in methanol-*d*_4_, which shift to δ −23.79 and δ −24.20 in dichloromethane-*d*_2_. These are important observations as it suggests that a typical SABRE complex of the form [Ir(H)_2_(IMes)(**A** or **B**)_3_]Cl, which would give a single hydride ligand signal for the two chemically equivalent atoms, is not responsible for SABRE of **A** and **B**. Consequently, 2D NMR characterization and X-ray crystallography was used to confirm that these resonances belong to neutral [IrCl(H)_2_(IMes)(**A** or **B**)_2_]. This reflects a type of SABRE catalyst where hydride ligand inequivalence is achieved chemically rather than magnetically.

### Reaction Time Course Studies to Unravel the Formation of [IrCl(H)_2_(IMes)(A or B)_2_]

SABRE catalysts have been reported to form from **1** via the ready displacement of its Cl ligand to form [Ir(η^**2**^-η^**2**^-COD)(IMes)(substrate)]Cl, which subsequently adds H_2_ to form [Ir(H)_2_(IMes)(substrate)_3_]Cl via the intermediate [Ir(H)_2_(η^**2**^-η^**2**^-COD)(IMes)(substrate)]Cl.^13,41^ Experiments were performed here to investigate the reaction pathway leading to [IrCl(H)_2_(IMes)(substrate)_2_] with the electron-deficient dihalogenated pyridines. Examination of solutions of **1** and **A** confirmed incomplete halide substitution to give [Ir(**A**)(η^**2**^-η^**2**^-COD)(IMes)]Cl (**2**_**A**_), indicated by new ^1^H NMR resonances for **2**_**A**_ (see the Supporting Information S3.1). The equilibrium position between **1** and **2**_**A**_ was probed between 248 and 303 K and the corresponding Van’t Hoff plot (the Supporting Information S3.1 and Figure S6) allowed Δ*H*^Θ^ and Δ*S*^Θ^ to be determined as −14.36 ± 0.7 kJ mol^–1^ and −81.2 ± 2.3 J K^–1^ mol^–1^ respectively. Taken together, these give a Δ*G*^Θ^ of +9.4 ± 1.4 kJ mol^–1^ at 298 K in accordance with the lower stability of **2**_**A**_. The increase in order (negative entropy change) observed on moving to **2**_**A**_ indicates increased solvation for **2**_**A**_ due to its charge, and possibly lower rotational freedom for its ligands. We note that with pyridine, the corresponding equilibrium position lies much more heavily in favor of the substitution product.^42,43^

**Figure 2:**
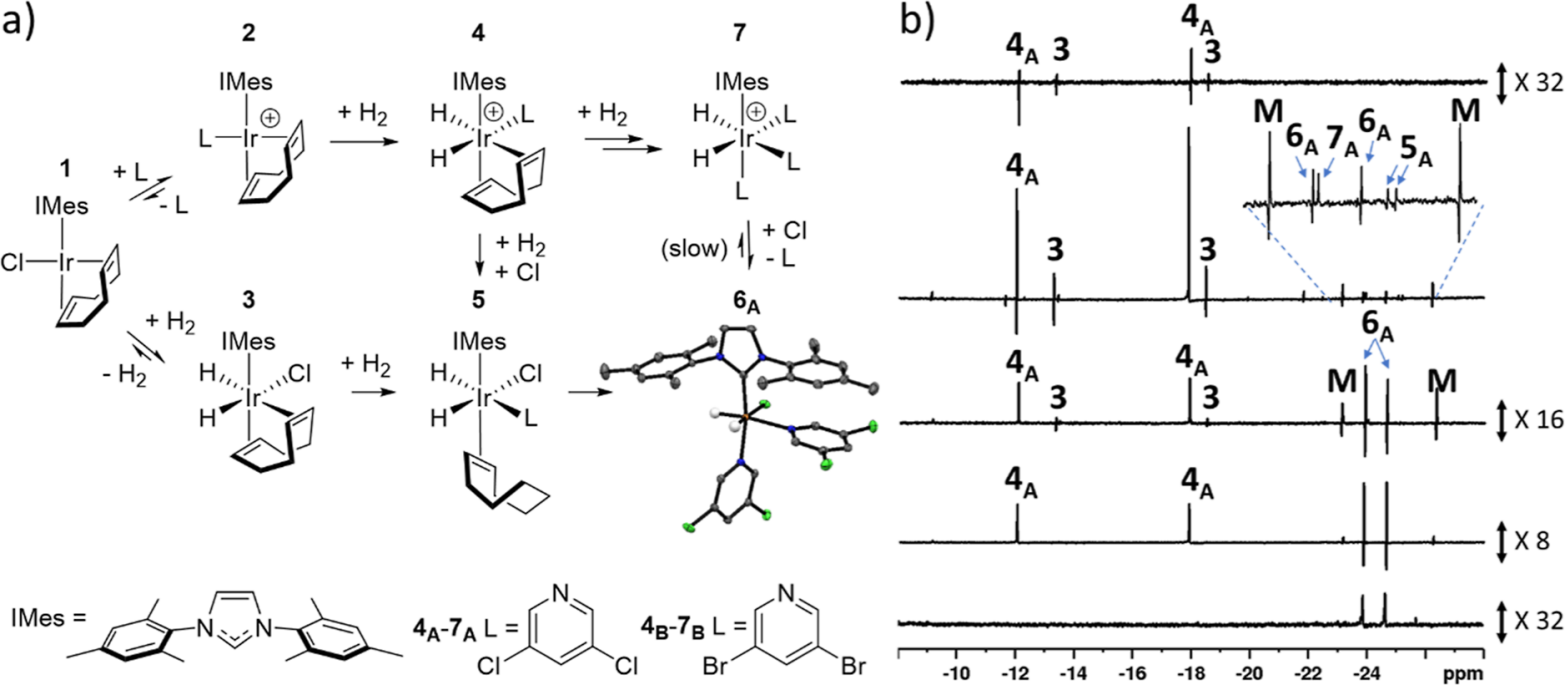
(a) Formation of SABRE active complexes **6** and **7** from the precatalyst **1**. For **2**_**A**_, **4**_**A**_, **5**_**A**_, **6**_**A**_, and **7**_**A**_ L is 3,5-dichloropyridine and for **2**_**B**_, **4**_**B**_, **5**_B_, **6**_**B**_, and **7**_**B**_ L is 3,5-dibromopyridine. The structure of **6**_**A**_ determined using X-ray crystallography is shown with thermal ellipsoids at 50% probability and all nonhydride hydrogen atoms omitted for clarity (gray is carbon, blue is nitrogen, white is hydrogen, green is chlorine and orange is iridium). The structure for **6**_**B**_ is given in the Supporting Information. (b) Representative single-scan ^1^H NMR spectra recorded at 243 K after addition of parahydrogen to an equilibrium mixture of **1** and **2**_**A**_ in methanol-*d*_4_. The spectra are recorded (upper to lower) *ca* 2 min, 20 min, 45 min, and a few hours after parahydrogen addition at 243 K. The lower spectrum is recorded at 298 K after the sample was warmed to room temperature and left for 2 days. Signals denoted as M correspond to a methanol or water-bound complex.

H_2_ addition to **1** and **2**_**A**_ might therefore be expected to form [Ir(Cl)(H)_2_(η^**2**^-η^**2**^-COD))(IMes)] (**3**) and [Ir(H)_2_(**A**)(η^**2**^-η^**2**^-COD)(IMes)]Cl (**4**_**A**_) respectively (Figure 2a). Accordingly, a dynamic mixture of **1** and **2**_**A**_ (ratio of *ca* 2:1) was cooled to 243 K and its reaction with *p*H_2_ in methanol-*d*_4_ monitored using a series of single scan ^1^H NMR spectra. In the first of these spectra, a strong pair of PHIP-enhanced hydride resonances were observed, as antiphase doublets at δ −12.01, δ− 17.89, alongside two smaller PHIP-enhanced hydride ligand signals at δ −13.45 and δ −18.50. As these relative signal intensities probe reaction flux they relate to the rate of H_2_ addition to **1** and **2**_**A**_ and their concentrations.^44^ Consequently, we can conclude that H_2_ addition to **2**_**A**_ is faster than **1**.

Careful temperature cycling of a separate sample, by warming from 243 to 263 K for *ca* 10 min, before being cooled again to 243 K, allowed the species yielding the signals at δ −12.01 and δ −17.89 to form in high proportion, and to be characterized by 2D NMR methods as anticipated **4**_**A**_ (see the Supporting Information S3.4). The minor signals at δ −13.45 and δ −18.50 arise from **3**, as confirmed by a control reaction where H_2_ adds to **1** alone in methanol-*d*_4_ at 243 K. Furthermore, the hydride ligand signals for **3** retain visible PHIP polarization for many minutes as a consequence of slow *p*H_2_ destruction in these samples. Careful studies involving further temperature cycling revealed that the dynamic equilibrium between **1** and **2**_**A**_ in solution responds to H_2_ addition to enable complete conversion to **4**_**A**_. The hydride ligand signals of **4**_**A**_ were probed by EXSY spectroscopy at 243–263 K and found to be inert to H_2_ exchange on the NMR time scale. This observation accounts for the decreasing PHIP signal gain of the hydride ligand signals for **4**_**A**_ as the reaction proceeds, as singlet order within **4**_**A**_ is not replenished by reversible *p*H_2_ exchange.

As dihydrides **3** and **4**_**A**_ both contain a diene ligand, they are intrinsically unstable to hydrogenation. Logically, [Ir(Cl)(H)_2_(**A**)(η^2^-COE)(IMes)] (**5**_**A**_) would result after partial COD hydrogenation, and ultimately [Ir(Cl)(H)_2_(**A**)_2_(IMes)] (**6**_**A**_) or [Ir(H)_2_(**A**)_3_(IMes)]Cl (**7**_**A**_) form after further reaction (Figure 2a). Two PHIP-enhanced resonances, proposed to arise from **5**_**A**_, were located at δ −24.49 and δ −24.59 at 243 K. Full characterization data for **5**_**A**_ could not be collected due to its very high reactivity and low concentration, which prevents its detection without hyperpolarization. However, the observation that its hydride resonances are PHIP-enhanced, in conjunction with the fact **4**_**A**_ does not undergo rapid H_2_ exchange, suggest that the original hydride ligands of **4**_**A**_ become the two additional protons in the COE ligand of **5**_**A**_. This is supported by weak hyperpolarization of the ^1^H NMR signal of free cyclooctane at δ 1.6. Interestingly, upon heating to 253 K, the hydride ligand resonances attributed to **5**_**A**_ are no longer visible. Additionally, the conversion of 50% of **4**_**A**_ into **6**_**A**_ and **7**_**A**_ results over an 80 min period, leading to a **6**_**A**_/**7**_**A**_ ratio of 9:1. In contrast, when a methanol-*d*_4_ mixture of **1** and **2**_**A**_ is exposed to H_2_ at 298 K instead, the transient hydride ligand signals seen for **4**_**A**_ are now rapidly replaced by broad resonances for **6**_**A**_, at δ −24.10 and δ −24.72, and a weak singlet for **7**_**A**_ at δ −23.18. The ratio of **6**_**A**_/**7**_**A**_ proved ultimately to reach an equilibrium position of 16:1 at this temperature, which is unusual as previous studies with a wide array of pyridyl-containing substrates report chloride displacement to form species like **7**_**A**_ preferentially to **6**_**A**_.^41,42^ The fact the **6**_**A**_/**7**_**A**_ ratio changes over time suggests that the initial ratio results from kinetic control of the reaction , with slower equilibration leading to a thermodynamic preference for **6**_**A**_. At 298 K, the hydride signals for **6**_**A**_ are the most dominant in any hyperpolarized ^1^H NMR spectra recorded, and the resonances for its bound **A** ligand that lies *trans* to hydride, alongside those of free **A**, are enhanced by SABRE. Consequently, **6**_**A**_ reflects a novel neutral SABRE polarization transfer catalyst. It is important to note that at these higher temperatures relatively facile H–D exchange between H_2_ and methanol-*d*_4_, becomes evident, which complicates the appearance of the hydride ligand signals of **6**_**A**_ and **7**_**A**_; there is also evidence for deuteration of **A**.^33,34^

The equilibrium position between **6**_**A**_ and **7**_**A**_ can be tipped to favor the exclusive formation of **6**_**A**_ by changing the solvent to dichloromethane-*d*_2_. When *p*H_2_ addition is monitored at 253 K in dichloromethane-*d*_2_, a much slower reaction ensues, with signals again observed for **3**, **5**_**A**_ and **6**_**A**_ with relative PHIP signal intensities 1:0.45:5.3. Upon leaving this sample for 1 h at 298 K, prior to cooling for further characterization, **6**_**A**_ proved to form cleanly as revealed by the detection of NMR signals at δ −23.98 and δ −24.68. These two hydride ligand resonances move to δ −23.96 and δ −23.99 at 243 K such that they are separated by 26 Hz (11.73 T) and therefore they exhibit chemical shifts that are highly temperature dependent. In fact, upon cooling further to 233 K, they become reflective of an A_2_ spin system, while at 228 K their relative positions invert and return to AB behavior with a chemical shift difference of *ca* 24 Hz (see the Supporting Information S3.5 and Figure S14 and Table S12). Thus, the two hydride resonances for **6**_**A**_ can reflect AB, A_2_ or AX type spin systems depending on the temperature.

Collectively, of the dominant H_2_ addition products formed via **1** and **2**_**A**_, five have been assigned as **3**, **4**_**A**_, **5**_**A**_, **6**_**A**_, and **7**_**A**_, although a minor species giving rise to signals at δ −23.24 and δ −26.14 could not be attributed. An adduct-containing methanol-*d*_4_ or H_2_O would be supported by their similar chemical shifts^45^ and the fact these signals are not observed in dichloromethane-*d*_2_. The thermodynamic product in both methanol and dichloromethane is therefore **6**_**A**_. Excitingly, this was confirmed from X-ray crystallography (Figure 2a). These structures are notable as they reflect a rare example of an X-ray structure for a SABRE active complex, which are usually characterized exclusively by 2D NMR.

Analogous reactions occur with 3,5-dibromopyridine (**B**) leading to similar complexes. Briefly, **B** reacts with **1** in methanol-*d*_4_, to form an equilibrium with [Ir(**B**)(IMes)(η^**2**^-η^**2**^-COD)]Cl (**2**_**B**_) (see the Supporting Information, Section S3.7). However, now Δ*H*^Θ^ and Δ*S*^Θ^ for the equilibrium between **1** and **2**_**B**_ are smaller than those for **1** and **2**_**A**_ (−16.4 ± 0.6 kJ mol^–1^ and −86.2 ± 1.7 J K^–1^ mol^–1^ respectively). Taken together, these give a Δ*G*^Θ^ of +9.3 ± 1.1 kJ mol^–1^ at 298 K in accordance with the lower stability of **2**_**B**_ compared to **1**. These data suggest that the interaction of more electron deficient **A** with the metal center is linked to a smaller release of energy and consequently a weaker Ir–**A** bond in **2**_**A**_, when compared to Ir–**B** in **2**_**B**_. Addition of H_2_ to equilibrium mixtures of **1** and **2**_**B**_ at 248 K reveals the slow formation of analogous **4**_**B**_ (characterization data is presented in the Supporting Information, Table S15), with **3** remaining as a minor product. Subsequently, signals for **5**_**B**_ and **6**_**B**_ grow in size in accordance with their formation from **4**_**B**_. **6**_**B**_ is also deduced to form as the thermodynamic product in direct analogy with **6**_**A**_. A mixture of **A** and **B** was also examined, now the size of the PHIP hydride ligand signals for **4**_**A**_ and **4**_**B**_ link to the proportion of the parent **2**_**A**_ and **2**_**B**_. These data revealed that the signals for **4**_**A**_ were ∼11% larger than those for **4**_**B**_, based on equivalent amounts of the precursor at 253 K. Hence **2**_**A**_ undergoes slightly faster H_2_ addition than **2**_**B**_.

### Improving Polarization by Doping with Sulfoxides

**Figure 3:**
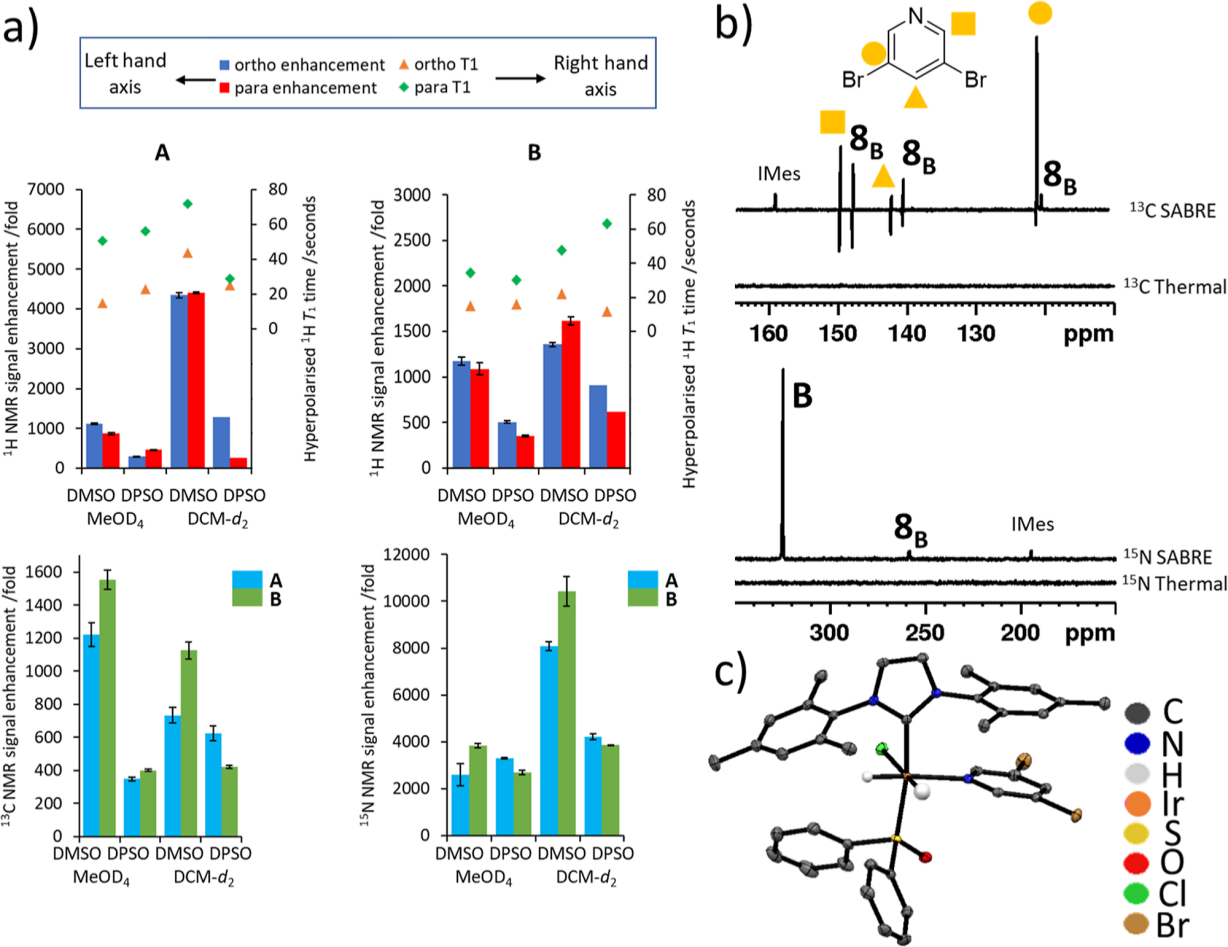
(a) Summary of ^1^H NMR signal enhancements for **A** (upper left) and **B** (upper left) and *meta*^13^C (lower left) and ^15^N (lower right) NMR signal enhancements achieved using different conditions (either DMSO or DPSO coligand in either methanol-*d*_4_ or dichloromethane-*d*_2_) (b) representative SABRE hyperpolarized ^13^C (upper) and ^15^N (lower) NMR spectra when **1** (5 mM), DMSO (25 mM) and **B** (10 50 mM) are shaken with 3 bar *p*H_2_ for 10 s at 1 μT in methanol-*d*_4_ (upper) and dichloromethane-*d*_2_ (lower). Enhanced signals for the IMes ligand bound in **8**_**B**_ are marked. Analogous thermally polarized spectra are shown below the hyperpolarized counterpart. (c) Structure of **9**_**B**_ determined from X-ray crystallography. All nonhydride hydrogen atoms and solvent of crystallization have been removed for clarity and thermal ellipsoids are shown at 50% probability.

Recently, it has been shown that coligands can play a key role in SABRE as they facilitate coordination of weakly ligating or sterically large substrates, thereby allowing them to become hyperpolarized.^21,23,46^ As **A** and **B** are weakly ligating substrates (typical catalysts of the form [Ir(H)_2_(IMes)(substrate)_3_]Cl are not the dominant product), a series of samples were prepared to examine if the resulting NMR polarization level could be improved using the coligands dimethylsulfoxide (DMSO) or diphenylsulfoxide (DPSO). For these experiments, the substrate concentration was arbitrarily fixed at 50 mM and that of the coligand at 25 mM. When SABRE experiments on **A** and **B** are performed under these conditions in either methanol-*d*_4_ or dichloromethane-*d*_2_, the improvement in signal enhancement is dramatic. For example, the ^1^H NMR signal enhancements for **A** seen using DMSO as a coligand become 1119 ± 14 and 870 ± 28-fold for the *ortho* and *para* sites of free **A,** respectively. These values are five times larger than the analogous 205 ± 4 and 160 ± 3-fold achieved without any DMSO present. This improvement is even more substantial in dichloromethane-*d*_2_ where these enhancements become 4350 ± 65 and 4413 ± 22-fold, reflecting a *ca* 100 times gain in efficiency compared to that seen without DMSO. Substantial, but less dramatic, effects are observed for **B**, as its NMR signal enhancements can be increased from 165 ± 5- and 134 ± 4-fold to 1172 ± 42- and 1088 ± 68-fold in methanol-*d*_4_ and from 17 ± 1 and 20 ± 1-fold to 1355 ± 21 and 1613 ± 46-fold in dichloromethane-*d*_2_ with DMSO. The ^1^H NMR signal enhancements of both **A** and **B** can also be improved by using bulkier DPSO as a coligand, but now the overall ^1^H NMR signal enhancements are *ca* 2–4 times lower than those achieved using DMSO (Figure 3 and the Supporting Information S4 and Tables S18 and S19).

The higher SABRE efficiency when a sulfoxide catalyst is used therefore suggests that the polarization transfer catalyst is more efficient at catalyzing the transfer of spin order from *p*H_2_-derived hydride ligands into free substrate. 2D NMR experiments were used to characterize the structure of these catalysts, which are of the form [IrCl(H)_2_(IMes)(**A** or **B**)(DMSO)], **8**, or [IrCl(H)_2_(IMes)(**A** or **B**)(DPSO)], **9**, depending on the specific coligand used (see the Supporting Information S5). Notably, an X-ray crystal structure of **9**_**B**_ was obtained.

When PHIP reaction time courses are monitored at 245 K in methanol-*d*_4_ for these preparations (see the Supporting Information S5.6), the presence of sulfoxide does not appear to influence the equilibrium connecting **1** and **2**_**A**_ (i.e., analogues of **2**_**A**_ and **3** containing DMSO instead of L or Cl are not observed). **6**_**A**_ and **7**_**A**_ are still observed to form competitively though, and any signals for **5** are masked by the strong broad signals for **6**_**A**_. Furthermore, while signals for **8**_**A**_ are seen, those of the methanol adduct are not, and a new species is detected at δ −14.45 and −26.24. This species is transient and its chemical shifts are consistent with it being an analogue of **5**_**A**_ whereby the substrate has been swapped for DMSO. Consequently, it is deduced that these sulfoxide-containing SABRE catalysts can form by replacement of the substrate for DMSO in **5** and subsequent loss of COE. Slower equilibration between **6**_**A**_, **7**_**A**_, and **8**_**A**_ occurs to form **8**_**A**_ almost exclusively. No DMSO analogue of **4**_**A**_ is detected.

The SABRE efficiency of these species follows the order **8** > **9** > **6**. The corresponding hyperpolarized ^1^H *T*_1_ measurements indicated that relaxation times for hyperpolarized spins remain broadly similar in the presence of a coligand (compare Figures 3a and 1c). Consequently, the higher SABRE efficiency of **8** and **9** must be rationalized through differences in ligand exchange rate when compared to **6**. Accordingly, the rate of dissociation of **A** was measured in **6**_**A**_ and **8**_**A**_, and found to be significantly slower in **8**_**A**_ (see the Supporting Information S6). At 288 K, this rate was too fast to be measured by EXSY for **6**_**A**_, whereas in **8**_**A**_ it was 6.74 ± 0.02 s^–1^ in methanol-*d*_4_ and 5.44 ± 0.36 s^–1^ in dichloromethane-*d*_2_. These values are close to the optimum predicted substrate exchange rate^47^ of *ca* 4.5 s^–1^ for pyridine which explains the high performance of **8**_**A**_ compared to **6**_**A**_. Moreover, the higher ^1^H NMR signal enhancements of **A** in dichloromethane-*d*_2_ can be rationalized in terms of the beneficial slower substrate dissociation rate when compared to methanol-*d*_4_. Interestingly, the Δ*H*^‡^ values for substrate dissociation from both **6**_**A**_ and **8**_**A**_ were found to be similar (88 ± 2 kJ mol^–1^ for **6**_**A**_, 86 ± 1 kJ mol^–1^ for **8**_**A**_ in dichloromethane-*d*_2_ and 84 ± 1 kJ mol^–1^ for **8**_**A**_ in methanol-*d*_4_, respectively, see Supporting Information S6). Hence, the promoting force for these exchange rate differences is Δ*S*^‡^, which is much larger in **6**_**A**_ (105 ± 8 J K^–1^ mol^–1^ in dichloromethane-*d*_2_) compared to **8**_**A**_ (73 ± 3 and 60 ± 4 J K^–1^ mol^–1^ in dichloromethane-*d*_2_ and methanol-*d*_4_, respectively). The difference in behavior of **B**, when comparing **6**_**B**_ and **8**_**B**_, is also driven by entropy. The dissociation rate from **8** is 15% larger for **A** than **B** at 288 K, and the ^1^H NMR signal enhancements of **A** are *ca* 2.5 times higher than **B**. However, the rate of ligand dissociation from **8**_**B**_ in methanol-*d*_4_ is 4.7 times slower than the analogous rate in **8**_**A**_ at 288 K, although their ^1^H SABRE performance is similar. The propagating hydride–pyridyl proton coupling involved in mediating SABRE transfer is around 1.2 Hz in species like [Ir(H)_2_(pyridine)_3_(IMes)]Cl.^48^ Similar measurements were conducted here and the analogous couplings were found to be ∼1.0 Hz, although loss of spin coherence during the long evolution time scale prevented their precise quantification. Nevertheless, it is clear that the order of magnitude improvement in ^1^H NMR signal enhancements achieved using **8** compared to **6** is due to the change in ligand exchange rate. When **9**_**B**_ was examined in dichloromethane-*d*_2_ the corresponding ligand loss rate at 288 K (1.31 ± 0.08 s^–1^) is very close to that of **8**_**B**_ in methanol which means that similar SABRE performance might be expected. However, **9** appeared to provide less efficient SABRE than **8** which is likely a result of the sterically bulkier DPSO giving rise to small (5–35%) proportions of **6**. We have shown that **6** has much faster ligand exchange, and therefore it is likely that it rapidly destroys *p*H_2_ spin order and its formation should be minimized. Accordingly, solutions containing sterically smaller DMSO form **8** almost exclusively, with no **6** discerned. This highlights how the steric and electronic properties of the sulfoxide are important factors in determining the formation and efficiency of SABRE catalysts.

There is also a dramatic improvement in ^13^C and ^15^N detectability using SABRE with sulfoxide, and while the *meta* carbon still receives the largest share of ^13^C polarization, the overall improvement allows signals for the *ortho* and *para* sites to be readily detected. Furthermore, as detailed in the Supporting Information, *ortho* and *meta* site polarization levels can become comparable. Collectively, the trends in ^1^H polarization level follow those for ^13^C and ^15^N polarization, with the coligand DMSO outperforming DPSO (Figure 3a). The largest ^13^C NMR signal enhancement for the *meta* carbon of 1553 ± 59-fold resulted for **B**, with the highest value for **A** being slightly lower at 1221 ± 70-fold. The enhanced ^13^C NMR signal of the *meta* resonance still appears predominantly as a singlet, indicative of direct SABRE–SHEATH transfer, although the minor antiphase component (Figure 3b) indicate competing transfer via a ^1^H site in **A** or **B** during movement to the magnet.^44,49^ As expected, the antiphase effect is much clearer for the enhanced ^13^C NMR signals of the *ortho* and *para* sites of **A** and **B**, where it is now reflective of the dominant polarization transfer mode.

Similarly, the ^15^N NMR signal enhancements attained for **A** and **B** were much higher than those recorded without a coligand. For example, 8086 ± 180-fold and 10,422 ± 632-fold signal enhancements respectively were recorded when samples were shaken at 6 μT. However, it appears that the SABRE performance of **8** has a different dependence on polarization transfer field than **6**, with most efficient ^15^N polarization resulting at 2 μT rather than 6 μT (see the Supporting Information, Figure S19a,b). Hence, unlike ^1^H transfer where a 6.5 mT transfer field is commonly optimal, there is a need for careful assessment when examining ^15^N transfer.^9,15,16,35^ The highest single scan ^15^N polarization levels achieved for **A** and **B** under optimized conditions at a 25 mM substrate concentration using **8** were 46,593 ± 1076-fold (15.4 ± 0.4% polarization) and 25,716 ± 1373-fold (8.5 ± 0.5% polarization), respectively.

As a further test, deuteration of the catalyst (IMes and DMSO ligands) has previously been shown to enable increased substrate polarization.^14^ However, in the present situation this change did not improve ^1^H, ^13^C, or ^15^N signal enhancements. This suggests that the long relaxation times are not significantly changed by catalyst deuteration. Consequently, SABRE polarization of these substrates can be completed with readily available starting materials. It is notable that **8** and **9** can also catalyze transfer of spin order from *p*H_2_ to the carbene ^13^C site (*ca* δ 160) and the imidazole rings ^15^N sites (*ca* δ 195, Figure 3b). In fact, the ^13^C and ^15^N NMR signal enhancements seen for these sites reached 178 ± 17 (**8**_**A**_) and 2615 ± 3-fold (**9**_**A**_), respectively (see the Supporting Information, Tables S19 and S20). Such effects are not typical when catalysts of the form [Ir(H)_2_(NHC)(Sub)_3_]Cl are examined. They are visible here in **8** and **9** due to their high intrinsic SABRE performance and the fact they contain inequivalent hydride ligands which results in different hydride ligand couplings to them, a requirement to unlock polarization transfer.

### Effects of RASER on ^1^H NMR Spectra

**Figure 4:**
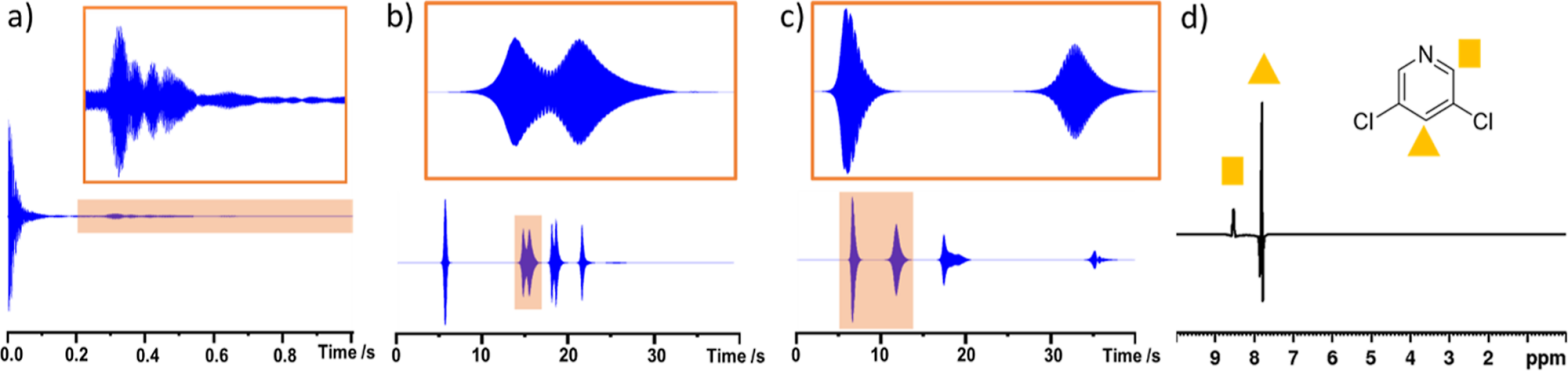
(a) ^1^H Free induction decay after a hyperpolarized sample containing **1** (5 mM), DMSO (25 mM), **A** (50 mM) and *p*H_2_ (3 bar) in methanol-*d*_4_ is excited using a 90° pulse. (b) ^1^H free induction decay with a longer acquisition time after a hyperpolarized sample containing **1** (5 mM), DMSO-*d*_6_ (25 mM) and **A** (100 mM) and *p*H_2_ (3 bar) in dichloromethane-*d*_4_ is excited using a 1° pulse (c) ^1^H free induction decay recorded with no radiofrequency excitation using the sample from (b). (d) ^1^H NMR spectra recorded without radiofrequency excitation (1 s acquisition) using the sample in (a). All spectra are recorded at 9.4 T and are not to the same vertical scale. Parts of the FID, colored in orange, are shown expanded in the orange inserts.

In some of the hyperpolarized ^1^H NMR spectra recorded using **8**, in which the highest ^1^H polarization levels can be achieved, interpretation of the NMR spectra proved to be complicated by the presence of baseline distortions. When the free induction decay of these examples are examined more closely they were often found to contain unusual features indicative of RASER effects (Figure 4a). These effects arise when a molecule spontaneously emits radio waves, and has been observed for molecules hyperpolarized using *p*H_2_.^24,26,27,50^ The RASER effects produced by **8** are obvious when a ^1^H NMR spectrum is recorded with a low flip angle as this leads to a FID envelope with much lower intensity (Figure 4b). In fact, when such samples are examined by simply recording a free induction decay without applying any radiofrequency excitation, the RASER response is clear (Figure 4c), and Fourier transformation yields signals for the *ortho* and *para* protons of **A** or **B** (Figure 4d). This confirms the origin of the signal distortions is the RASER effect. The observation of such effects is still unusual, but as SABRE is optimized the number of examples will grow.^24,26,27,50^

### Using SABRE-Enhanced NMR for Reaction Monitoring

This paper ends by demonstrating how the enhanced NMR signals of **A** can be used for reaction monitoring. For this a solvent-dependent methylation reaction was selected. Consequently, the hyperpolarization of **A** and **B** by **8** was tested in dioxane, THF, and THF-*d*_8_. Of these, the ^1^H NMR signal gains proved highest in THF-*d*_8_ (1740 ± 294-fold and 1081 ± 314-fold for the *ortho* and *para* sites of **A**, respectively, and 1892 ± 444 and 2135 ± 37 for the analogous sites in **B**). ^1^H SABRE efficiency under these conditions following the order dioxane < THF < THF-*d*_8_ < methanol-*d*_4_/dichloromethane-*d*_2_. These differences linked to substrate *T*_1_ in the different solvents (see the Supporting Information S7), although differences in ligand exchange rate will play a role. Similar trends were observed for the analogous ^13^C and ^15^N NMR signal gains.

**Figure 5:**
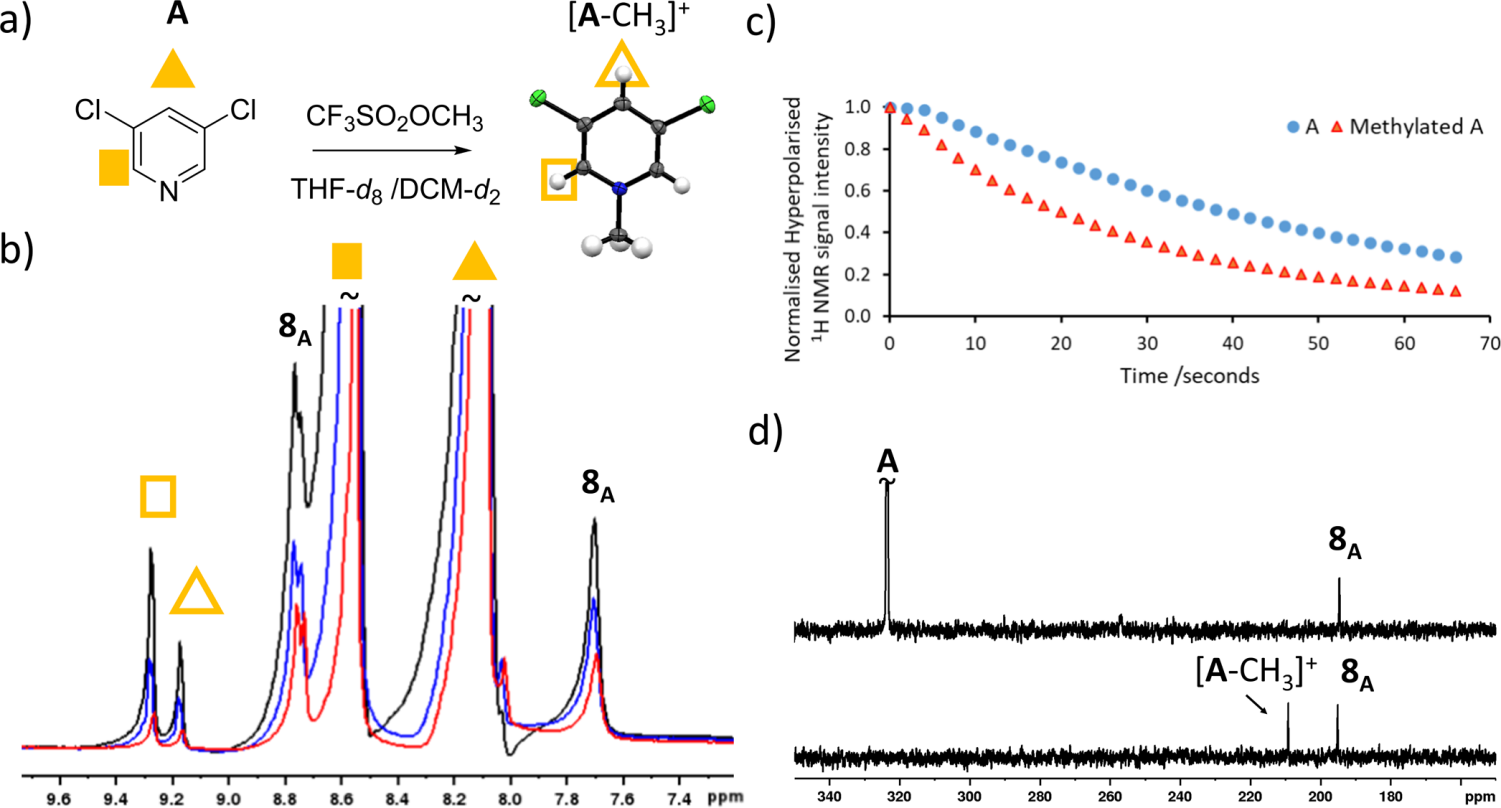
(a) Reaction of **A** with the methylating agent CF_3_SO_2_OCH_3_, the methylated product is confirmed from X-ray crystallography. (b) Example hyperpolarized ^1^H NMR spectra recorded after CF_3_SO_2_OCH_3_ (50 mM) is added to a sample of hyperpolarized **A** in THF-*d*_8_. **A** is hyperpolarized by shaking a solution of **1** (5 mM), DMSO (25 mM) and **A** (50 mM) with 3 bar *p*H_2_ for 10 s in the stray field of a 9.4 T magnet. After this process the lid is removed and CF_3_SO_2_OCH_3_ is added. Spectra are single-scanned and are recorded with a 5° flip angle. These three examples are taken 20 s (black), 30 s (blue) and 50 s (red) after the spectral acquisition commenced. (c) The signal intensities of reactant and product in hyperpolarized ^1^H NMR spectra over the time course of the spectral acquisition. Each data point took 2 s to acquire and each is recorded immediately after the previous data point. Time *t* = 0 corresponds to the starting point of spectral acquisition, which is *ca* 5–10 s after addition of CF_3_SO_2_OCH_3_. Note that each signal is normalized to its intensity in the first spectrum. (d) Example hyperpolarized ^15^N NMR spectra recorded for **A** by shaking a solution of **1** (5 mM), DMSO (50 mM) and **A** (250 mM) with 3 bar *p*H_2_ for 10 s at 2 μT in DCM-*d*_2_ before CF_3_SO_2_OCH_3_ addition (upper) and immediately after (lower) reshaking with *p*H_2_ and addition of CF_3_SO_2_OCH_3_ (250 mM). Both spectra recorded with a single 90° pulse. The signal at δ 195 is the IMes ligand in **8A**.

Immediately after the hyperpolarization of **A** in either THF-*d*_8_ or dichloromethane-*d*_2_, the lid of the NMR tube was removed and an equimolar solution of the methylating agent CF_3_SO_2_OCH_3_ added (Figure 5a). The sample was then placed into the spectrometer and a series of single-scan ^1^H NMR spectra with 5° flip angles collected. These spectra showed enhanced resonances for the reactant **A**, at δ 8.55 and 8.09, and for **A** bound in **8**_**A**_ at δ 8.77 and 7.71, which all decrease in intensity due to a combination of relaxation and magnetization depletion by the 5° pulses (Figure 5b,c). However, additional hyperpolarized signals for the reaction product *N*-methyl-3,5-dichloropyridine are visible at δ 9.28 and 9.18. These signals are indicative of the reaction of **A** with CF_3_SO_2_OCH_3_, which methylates the nitrogen center and was confirmed by both mass spectrometry and X-ray crystallography. Enhanced ^1^H NMR signals for this product reach just 2% of the intensity of those of **8**, and decrease in size until they become invisible as they relax back to their Boltzmann-derived signal intensity (Figure 5b,c). When this reaction is examined using ^15^N NMR, an enhanced signal for the methylation product is detected at δ 209 in DCM-*d*_2_ in a single scan measurement with 90° flip angle (Figure 5d). To detect such reaction products using naturally abundant ^15^N NMR with a 250 mM starting material loading would be effectively impossible. It is notable that as the reaction product now contains a methylated pyridine unit, it can no longer bind to the SABRE catalyst and cannot be hyperpolarized itself. It must therefore form directly from hyperpolarized **A**. These experiments collectively show that **8** can deliver NMR signal enhancements for **A** sufficient to allow organic reaction products to be detected. This method may therefore find use in monitoring chemical reactions in real time, or detecting low concentration reaction products. Excitingly, the long ^1^H *T*_1_ time for **A** at 9.4 T does not necessitate use of heteronuclei or long-lived singlet states^39,51^ to lengthen the time window over which measurements can be made.

## Experimental Section

All NMR measurements were carried out on a 400 MHz Bruker AVANCE III spectrometer at 298 K unless otherwise stated. *Para* hydrogen (*p*H_2_) was produced by passing hydrogen gas over a spin-exchange catalyst (Fe_2_O_3_) at 28 K and used for all hyperpolarization experiments at 3 bar overpressure. This method produces constant *p*H_2_ with *ca* 99% enrichment. ^1^H (400 MHz) and ^13^C (100.6 MHz) NMR spectra were recorded with an internal deuterium lock. Chemical shifts are quoted as parts per million and referenced to residual solvent. ^13^C and ^15^N NMR spectra were recorded with broadband proton decoupling. Coupling constants (*J*) are quoted in Hertz. All starting compounds were purchased from Sigma-Aldrich, Fluorochem, or Alfa-Aesar and used as supplied without further purification. **1**(52) and **1**-*d*_**24**_^37^ were synthesized in our laboratory according to literature procedures. The shake-and-drop method was employed for recording hyperpolarized SABRE NMR spectra. Samples were prepared in a 5-mm NMR tube that was fitted with a J. Young’s tap. The iridium precatalyst used was [IrCl(COD)(IMes)] [where IMes = 1,3-bis(2,4,6-trimethyl-phenyl)imidazole-2-ylidene and COD = cis,*cis*-1,5-cyclooctadiene]. The NMR samples were subsequently degassed by two freeze–pump–thaw cycles using a Schlenk line before filling the tube with H_2_. Upon reaction for 2–3 h, the H_2_ atmosphere was replaced with *p*H_2_ and the tubes were shaken vigorously for 10 s at the required polarization transfer field. Immediately after that, the NMR tubes were put inside the spectrometer for immediate NMR detection. Polarization transfer fields of 6.5 mT are achieved in the stray field of our 9.4 T magnet, whereas fields of 1–6 μT are achieved by using a mu metal shielded solenoid.^21^ The direction of this field is aligned with the direction of the spectrometer magnet such that the sample does not cross through a null point. Therefore, all SABRE experiments are performed under ALTADENA-like conditions where the hyperpolarization step takes place outside the magnet. For hyperpolarized measurements at low temperature, samples were cooled to the indicated temperature inside the NMR spectrometer. The sample was then removed and *p*H_2_ added before being shaken at 65 G and rapidly inserted into the spectrometer. Even though the *p*H_2_ addition and shaking process are performed at room temperature, the sample is only outside of the spectrometer for roughly 30 s, and any warming is assumed insignificant. Spectral acquisition commenced immediately after sample insertion using a 45° pulse. The conditions are likely a mixture of both ALTADENA and PASADENA. In the first experiment it is likely to be ALTADENA, although this effect should vanish and become PASADENA in subsequent measurements as hydrogenation continues in the spectrometer field (9.4 T).

NMR signal enhancements were calculated by dividing the hyperpolarized integral intensity by the corresponding intensity from a 1 scan thermal recorded and processed under the same conditions. Both hyperpolarized and thermally polarized spectra were recorded on the same sample using the same spectrometer settings. ^13^C and ^15^N NMR signal enhancements were calculated by reference to the thermally polarized solvent (for ^13^C) or a standard solution of ^15^NH_4_Cl and are calculated according to equations previously reported.^44^ Hyperpolarized ^1^H *T*_1_ times were measured by recording a series of consecutive single scan ^1^H NMR spectra with low flip angle. The corresponding integral intensities were fit to a series of equations accounting for rf excitation and relaxation to allow the later to be determined, more information is given in the Supporting Information S2. Substrate exchange rates were measured using exchange spectroscopy,^44^ more information is given in the Supporting Information S6. For X-ray crystallography, suitable crystals were selected and mounted on an Oxford-Diffraction SuperNova dual-source X-ray diffractometer equipped with copper and molybdenum sources and a HyPix-6000HE detector. Cooling to 110 K was achieved using an Oxford Instruments Cryojet. The details of the structural refinement and key parameters of the unit cell(s) are given in the Supporting Information.

## Conclusions

This work has shown that the electron deficient N-heterocycles 3,5-dichloropyridine and 3,5-dibromopyridine can be hyperpolarized using reversible polarization transfer from *para* hydrogen. This was achieved using catalysts of the form [IrCl(H)_2_(IMes)(substrate)_2_] (**6**) or [IrCl(H)_2_(IMes)(sulfoxide)(substrate)] (**8** or **9**) where hydride ligand symmetry is broken through chemical inequivalence. Notably, the preference for **6** is unusual, as charged species of the type [Ir(H)_2_(IMes)(substrate)_3_]Cl with magnetically distinct hydride ligands are most commonly formed when pyridyl-derived substrates are examined. This reflects the low basicity of these agents, and is supported by ligand exchange studies that revealed **6** was too reactive for optimal SABRE. The sulfoxide-containing catalysts **8** or **9** proved to be more stable than **6**, and reflect better SABRE catalysts. Ultimately, catalyst decomposition occurs after several days which is likely due to the formation of higher order iridium species, such as sulfoxide and/or sulfur-bridged dimers.^22,53^ Nonetheless, **8** delivers significant ^1^H, ^13^C and ^15^N NMR signal enhancements (up to 4350-, 1550- and 46,600-fold, or 14.0, 1.3, and 15.4% polarization) within seconds. One result of these strong signal gains was that both 3,5-dichloropyridine and 3,5-dibromopyridine now exhibit the RASER effect. Furthermore, enhanced ^1^H NMR signals exhibit very long *T*_1_ lifetimes (up to hundreds of seconds). This demonstrated that hyperpolarized ^1^H or ^15^N NMR signals of **A** can be used as a probe for reaction monitoring, in this case, its methylation.

This study has therefore demonstrated how utilization of the coligand DMSO can improve SABRE performance by an order of magnitude. In the future this improvement in catalytic efficiency is likely extendable to a wide range of other substrates, and many other classes of coligands may provide even greater improvements. This in turn enabled both ^13^C and ^15^N NMR detection of just 25 mM naturally abundant material in a single scan, which is of great benefit for the detection of low concentration pyridine derivatives using NMR. Furthermore, the long *T*_1_ values for these dihalopyridines enabled the examination of their methylation and detection of the RASER effect. Other pyridine reactivity could also be monitored which provide plenty of opportunity for developments in hyperpolarized reaction monitoring, particularly using low field or portable instruments.

## Data Availability

X-ray CIF files and raw NMR data collected as part of this study can be obtained via the link https://doi.org/10.15124/80e1a3d8-84a6-4194-b357-81c8883a56ea.

## Supporting Information Available

NMR and X-ray characterization data for complexes 1–9, additional PHIP reaction time courses, and ligand exchange studies (PDF)
X-ray CIF file for **6_A_** (CIF)
X-ray CIF file for **9_B_** (CIF)
X-ray CIF file for methylated **A** (CIF)
X-ray CIF file for **6_B_** (CIF)

## Supplementary Material

cs3c05378_si_001.pdf

cs3c05378_si_002.cif

cs3c05378_si_003.cif

cs3c05378_si_004.cif

cs3c05378_si_005.cif
