# Supplementary material for: Metal-Mediated Catalytic Polarization Transfer from para Hydrogen to 3,5-Dihalogenated Pyridines
Source: ACS Catal. 2024 Jan 5;14(2):994–1004. doi: 10.1021/acscatal.3c05378 (PMC10804365; doi:10.1021/acscatal.3c05378)
Supplement: Supplementary file 1 — cs3c05378_si_001.pdf [file cs3c05378_si_001.pdf]

Supporting Information

Metal-mediated catalytic polarisation transfer from *para*hydrogen to 3,5-dihalogenated pyridines

Ben. J. Tickner,[a,b] Marcus Dennington,[a,b] Benjamin G. Collins,[a,b,c] Callum A. Gater,[a,b] Theo F. N. Tanner,[b] Adrian C. Whitwood,[b] Peter J. Rayner,[a,b] Daniel P. Watts[c] and Simon B. Duckett[a,b*]

[a] Centre for Hyperpolarisation in Magnetic Resonance, University of York, Heslington, United Kingdom, YO10 5NY

[b] Department of Chemistry, University of York, Heslington, United Kingdom, YO10 5DD

[c] Department of Physics, Engineering and Technology, University of York, Heslington, United Kingdom, YO10 5DD

KEYWORDS SABRE catalysis; iridium; NMR; hyperpolarization; pyridine; parahydrogen

Corresponding authors email: simon.duckett@york.ac.uk

Table of Contents

S1: SABRE Hyperpolarisation of **A** and **B**
S2: Measurement of ^1H T_1 times
S3: Characterisation of metal complexes involved in SABRE
    S3.1: 2D NMR characterisation of equilibrium mixtures of **1** and $\mathbf{2_A}$
    S3.2: Hydrogen addition to equilibrium mixtures of **1** and $\mathbf{2_A}$
    S3.3: 2D NMR characterisation of $\mathbf{4_A}$
    S3.4: 2D NMR characterisation of **3**
    S3.5: 2D NMR characterisation of $\mathbf{6_A}$
    S3.6: X-Ray Crystallography of $\mathbf{6_A}$
    S3.7: 2D NMR characterisation of $\mathbf{2_B}$
    S3.8: 2D NMR characterisation of $\mathbf{4_B}$
    S3.9: 2D NMR characterisation of $\mathbf{6_B}$
    S3.10: X-Ray Crystallography of $\mathbf{6_B}$
S4: SABRE Hyperpolarisation of **A** and **B** in the presence of a sulfoxide coligand
S5: Characterisation of sulfoxide-containing metal complexes involved in SABRE
    S5.1: 2D NMR characterisation of $\mathbf{8_A}$
    S5.2: 2D NMR characterisation of $\mathbf{8_B}$
    S5.3: 2D NMR characterisation of $\mathbf{9_A}$
    S5.4: 2D NMR characterisation of $\mathbf{9_B}$
    S5.5: X-Ray Crystallography of $\mathbf{9_B}$
    S5.6: PHIP time-courses for formation of $\mathbf{8_A}$
S6: Ligand exchange rates in $\mathbf{6_A}$ and $\mathbf{8_A}$
S7: SABRE Hyperpolarisation of **A** and **B** in different solvents
S8: Methylation of 3,5-dichloropyridine

S1: SABRE Hyperpolarisation of A and B

Samples containing [IrCl(η²-η²-COD)(IMes) (**1**) (5 mM) and the substrate 3,5-dichloropyridine (**A**) or 3,5-dibromoopyridine (**B**) were dissolved in either methanol-d_4 or dichloromethane-d_2 (0.6 mL) and exposed to 3 bar H_2 gas for several hours to form SABRE-active iridium dihydride complexes. After this point, the H_2 atmosphere was replaced with pH$_2$ and a series of hyperpolarisation measurements were performed by shaking the sample for 10 seconds in the stray field of a 9.4 T spectrometer (*ca* 6.5 mT). NMR spectral acquisition commenced immediately after the sample was dropped into the spectrometer, and the hyperpolarisation process was repeated multiple times for each sample by replacing the spent pH$_2$ with fresh pH$_2$ before re-shaking the solution as described. Solutions were prepared with **A** and **B** at loadings of 5 and 10 equivalents relative to **1**.

^{1}H NMR signal enhancements for **A** and **B** are given in Tables S1 and S2 respectively. ^{1}H T_1 relaxation times are also given. ^{13}C and ^{15}N NMR signal enhancements for **A** and **B** are given in Tables S3 and S4 respectively.

Table S1: ^{1}H NMR signal enhancements and T_1 relaxation times for A at various loadings and in two solvents. The NMR signal enhancements are recorded by shaking a sample of [IrCl(η²-η²-COD)(IMes)] and A at the indicated loading and in the indicated solvent with 3-bar pH$_2$ for 10 seconds in the fringe field of a 9.4 T magnet (ca 6.5 mT). The relaxation times are measured from hyperpolarised samples (see section S2).

		25 mM **A**				50 mM **A**				100 mM **A**			
Solvent	Site	Ortho bound	Ortho free	Para free	Para bound	Ortho bound	Ortho free	Para free	Para bound	Ortho bound	Ortho free	Para free	Para bound
Methanol-d_4	^{1}H E/ fold	140 ± 8	237 ± 11	133 ± 6	80 ± 4	117 ± 14	205 ± 4	160 ± 3	62 ± 4	56 ± 1	108 ± 6	81 ± 4	47 ± 5
Dichloromethane-d_2	^{1}H E/ fold	132 ± 2	190 ± 8	176 ± 5	55 ± 3	35 ± 1	58 ± 2	38 ± 1	9 ± 1	18 ± 1	31 ± 2	25 ± 2	14 ± 1

Table S2:** **^{1}H NMR signal enhancements and T_1 relaxation times for B at various loadings and in two solvents. The NMR signal enhancements are recorded by shaking a sample of [IrCl(η^2-η^2-COD)(IMes)] and A at the indicated loading and in the indicated solvent with 3-bar pH_2 for 10 seconds in the fringe field of a 9.4 T magnet (ca 6.5 mT). The relaxation times are measured from hyperpolarised samples (see section S2).

		25 mM **B**				50 mM **B**				100 mM **B**			
Solvent	Site	Ortho bound	Ortho free	Para free	Para bound	Ortho bound	Ortho free	Para free	Para bound	Ortho bound	Ortho free	Para free	Para bound
Methanol-d_4	^{1}H E/ fold	58 ± 6	113 ± 8	97 ± 8	50 ± 4	228 ± 18	165 ± 5	134 ± 4	70 ± 2	83 ± 14	148 ± 24	111 ± 17	58 ± 4
Dichloromethane-d_2	^{1}H E/ fold	125 ± 11	272 ± 21	151 ± 13	90 ± 7	10 ± 1	17 ± 1	20 ± 1	6 ± 1	17 ± 1	37 ± 3	29 ± 2	19 ± 1

Table S3:** **^{13}C NMR signal enhancements for A and B at various loadings and in two solvents. The NMR signal enhancements are recorded by shaking a sample of [IrCl(η^2-η^2-COD)(IMes)] and A or B at the indicated loading and in the indicated solvent with 3-bar pH_2 for 10 seconds in a mu metal shield at 1 mG. Enhancements are for the free ligand signal.

	**A**			**B**		
	25 mM	50 mM	100 mM	25 mM	50 mM	100 mM
Methanol-d_4	110 ± 10 (*meta*)	61 ± 10 (*meta*)	41 ± 1 (*meta*)<br>10 ± 1 (*ortho*)<br>15 ± 1 (*para*)	243 ± 21 (*meta*)	91 ± 8 (*meta*)	29 ± 9 (*meta*)
Dichloromethane-d_2	61 ± 2 (*meta*)	21 ± 4 (*meta*)<br>23 ± 1 (*ortho*)<br>39 ± 9 (*para*)	12 ± 2<br>12 ± 1 (*ortho*)<br>21 ± 1 (*para*)	105 ± 2 (*meta*)	31 ± 1 (*meta*)	20 ± 2 (*meta*)

Table S4:** **^{15}N NMR signal enhancements for A and B at various loadings and in two solvents. The NMR signal enhancements are recorded by shaking a sample of [IrCl(η^2-η^2-COD)(IMes)] and A or B at the indicated loading and in the indicated solvent with 3-bar pH_2 for 10 seconds in a mu metal shield at 6 mG. Enhancements are for the free ligand signal.

	**A**			**B**		
	25 mM	50 mM	100 mM	25 mM	50 mM	100 mM
Methanol-d_4	No signal	2209 ± 903	4702 ± 91<br>(^{15}N T_1 of 36 .2 s)	2332 ± 258	7044 ± 961	5762 ± 179
Dichloromethane-d_2	402	2295 ± 271<br>45 ± 1 (bound)	5203 ± 387	809 ± 13<br>196 ± 75 (bound)	4422 ± 143	5077 ± 131

S2: Measurement of ^{1}H T_1 times

Immediately after shaking the solutions of the indicated SABRE catalyst with pH$_2$ (3 bar for 10 seconds in the stray field of a 9.4 T magnet), the sample was rapidly inserted into the 9.4 T spectrometer to record a hyperprolarised T_1. This involved the collection of a succession of single scan ^{1}H NMR spectra with a 5° pulse at 298 K that were separated by 7.5 s time intervals up to a 150-220 s time window. For the study investigating the effect of catalyst concentration on the T_1 time of **A** (Figure S1), flip angles of 10.35° and time spacings of 10 seconds were used, with a total measurement time window of 290 s.

The integral intensities of the ^{1}H NMR resonances for free **A** or **B**, and those bound to the SABRE catalyst (where applicable) were fitted to a model to extract a T_1. In this model the hyperpolarised signals of species X, S_X detected by the low flip angle pulse at time t is calculated according to Equation 1 where M_X is the magnetization of species X and θ is the flip angle. The magnetization of species X remaining after the pulse is given by Equation 2.

$$(S_X)_t = (M_X)_{t-\delta t} \sin\theta \qquad (\mathbf{1})$$

$$(M_X)_t = (M_X)_{t-\delta t} \cos\theta \qquad (\mathbf{2})$$

The magnetisation of species X changes during the time interval between pulses due to T_1 relaxation according to Equation 3. Note that this model does not account for changes in magnetisation of species X due to either chemical reaction (*i.e.* binding or unbinding to the iridium centre) or due to rehyperpolarisation during the time window of the T_1 measurement.

$$(M_X)_t = (M_X)_{t-\delta t} - \left(\frac{(M_X)_{t-\delta t}}{T_1}\right)\delta t \qquad (\mathbf{3})$$

T_1 times were calculated by fitting experimentally determined ^{1}H NMR integral intensities to values calculated using this model. Experimental integral intensities for each species were normalised to one at their highest intensity in the first spectra. T_1 times were calculated using Microsoft Excel to give the smallest squared difference between experimental and modelled integral intensities.

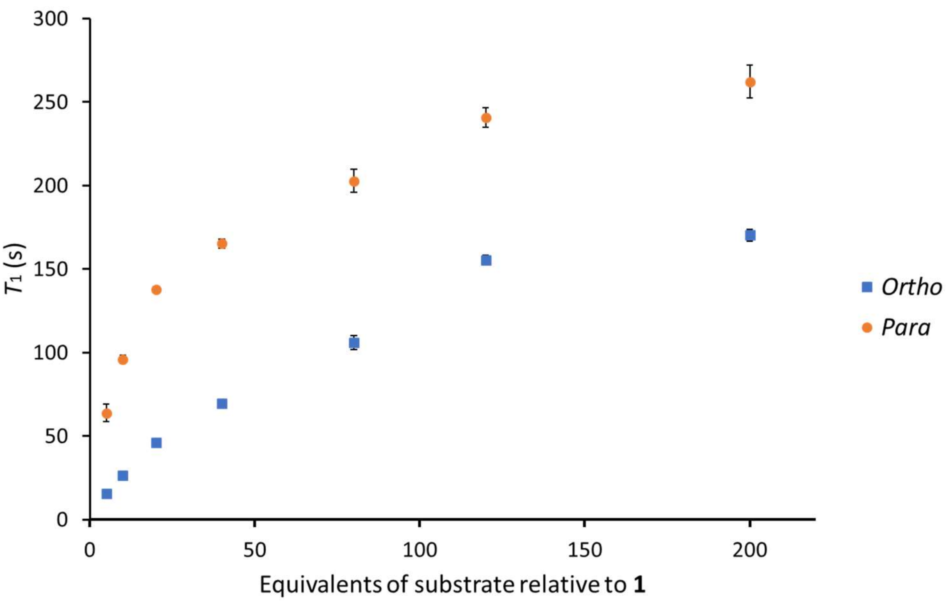

***Figure S1:** ^{1}H NMR signal T_1 times for A (50 mM) at the indicated loading relative to 1 in dichloromethane-d_2 at 9.4 T. Samples of different composition were prepared by fixing the concentration of A at 50 mM and adding the appropriate amount of 1.*

***Table S5:** ^{1}H NMR signal T_1 times for A (50 mM) at the indicated loading relative to 1 in dichloromethane-d_2 at 9.4 T. Each measurement series was repeated five times on the same sample, and the errors are one standard deviation of these five repeats.*

Equivalents of **A** (50 mM) relative to **1**	T_1 time (seconds) *ortho*	*para*
5	15.5 ± 1.8	63.9 ± 5.3
10	26.5 ± 1.2	96.0 ± 2.6
20	46.0 ± 0.3	138.0 ± 1.5
40	69.7 ± 1.7	165.4 ± 2.7
80	105.9 ± 4.2	202.8 ± 7.0
120	155.4 ± 3.0	240.8 ± 5.9
200	170.3 ± 3.6	262.3 ± 9.8

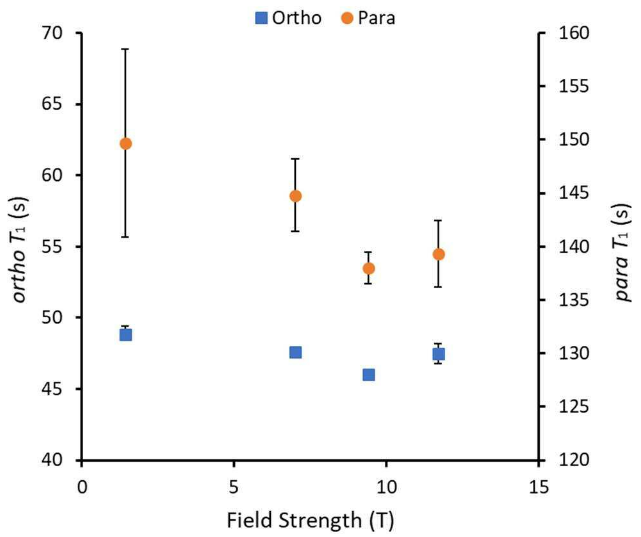

***Figure S2:** ^{1}H NMR signal T_1 times for A (50 mM) and 1 (2.5 mM) with 3 bar pH_2 in dichloromethane-d_2 (0.6 mL) at the indicated magnetic field.*

***Table S6:** ^{1}H NMR signal T_1 times for A (50 mM) and 1 (2.5 mM) with 3 bar pH_2 in dichloromethane-d_2 (0.6 mL) at the indicated magnetic field. Each measurement series was repeated five times on the same sample and the errors are one standard deviation of these five repeats. For the values at 1.41 T, a total measurement time window of 190 seconds was used with a flip angle of 12.6° (compared to 290 s and 10.35° for the other fields).*

Magnetic Field (T)	T_1 time (seconds) *ortho*	*para*
1.41	48.8 ± 0.6	149.7 ± 8.8
7	47.6 ± 0.3	144.8 ± 3.4
9.4	46 ± 0.3	138 ± 1.5
11.7	47.5 ± 0.7	139.3 ± 3.1

S3: Characterisation of metal complexes involved in the SABRE process

S3.1: Characterisation of equilibrium mixtures of 1 and 2_A

The SABRE-precatalyst, **1**, in methanol-d_4 was characterised, in the presence of **A**, accordingly. The ^{1}H NMR spectrum of the sample displays four resonances in the alkene region (δ 3 – δ 5) as shown in Figure S3. The signals at δ 3.10 and δ 4.02 are more intense than the signals at δ 3.36 and δ 3.79. They belong to the SABRE precatalyst **1**. Integration of all four resonances shows that the δ 3.10 and δ 4.02 resonances come from groups in the same complex as they share the same relative integral, and δ 3.36 and δ 3.79 signals are attributed to the same, but a different, complex.
A 2D NOESY experiment was used to identify signals from groups close in space to the protons in the alkene yielding resonances at δ 3.10 and δ 4.02. The signal at δ 3.10 showed an nOe to a signal at δ 2.31, which had a relative integral of *ca* 6, and an nOe interaction with another signal at δ 7.05, which had an integral of *ca* 2. The other alkene resonance (δ 4.02) did not share any of these nOe interactions. Hence, δ 2.31 is clearly the equivalent *ortho* methyl protons of IMes, and investigation of the nOe interactions to the group yielding the δ 7.05 signal revealed that this signal was due to the *meta* aromatic protons of IMes, as they showed an nOe to a signal at δ 2.38, attributed to the *para* methyl protons of IMes. Therefore, it is clear that one set of COD alkene protons are bound *trans* to IMes (δ 4.02), and the other *cis* to IMes (δ 3.10). A 2D NOESY experiment identified the imidazole protons of the IMes ligand (δ 7.25), which shared an nOe with the *ortho* methyl protons of IMes. This assignment was further confirmed by the singlet multiplicity, and a relative integral of *ca* 2, for the signal at δ 7.25.

Attributing the ^{1}H coupling partners of the COD alkene resonances involved 2D COSY experiments. The δ 3.10 signal coupled to δ 1.28 and δ 1.65, whilst the δ 4.02 signal coupled to δ 1.34 and δ 1.72, which reflects the alkyl resonances of COD. Analysis of the 2D NOESY spectrum for these alkyl signals identified their relative positions since the δ 1.65 resonance showed an nOe peak to δ 1.72 and δ 1.28, but not δ 1.34, whereas the δ 1.28 signal showed an nOe to δ 1.34 and δ 1.65 and not δ 1.72.

Signals for the substitution product, **2$_A$** were also visible: its alkene resonances at δ 3.79 and δ 3.36 are shifted from those of **1** (δ 3.10 and δ 4.02), suggesting the formation of a new complex with COD bound. An nOe interaction was observed between the signal at δ 3.79 and a doublet resonance with integral ca 2 at δ 7.87. A signal of this multiplicity and relative integral could only belong to the *ortho* protons of bound **A**. The *para* proton of 3,5-dichloropyridine (δ 8.19) was then assigned *via* its triplet multiplicity and mutual spin-spin coupling to the *ortho* protons of **A**. The other alkene signal in this complex (δ 3.36) showed an nOe connection to a resonance at δ 2.20, which had a relative integral of *ca* 6, and another nOe to a resonance at δ 7.16, which had a relative integral of *ca* 2. δ 2.20 was assigned to the *ortho* methyl protons of IMes and δ 7.16 to the aromatic *meta* proton of IMes. The 2D NOESY data also revealed an nOe interaction between the aromatic *meta* protons of IMes (δ 7.16) and an alkyl resonance with a relative integral of *ca* 3 at δ 2.46; this signal clearly belongs to the *para* methyl protons of IMes. The imidazole proton at δ 7.40 was identified *via* a nOe to only the ortho methyl protons at δ 2.20. COSY experiments revealed the alkyl coupling partners for the alkene signals of COD: δ 3.79 coupled to both δ 1.70 and δ 2.08, whilst δ 3.36 coupled to both δ 1.94 and δ 2.14. The ^{13}C chemical shifts reported in Table S7 were assigned through analysis of the cross-peaks in HMQC experiments. However, the cross-peaks for the COD alkyl protons overlapped significantly and were not be assigned unequivocally.

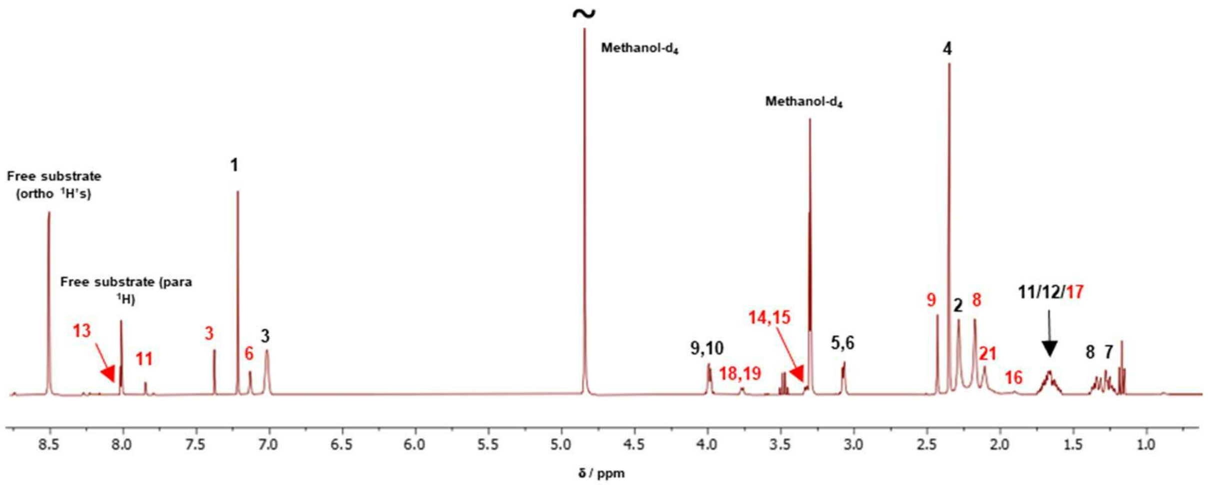

Figure S3: ^{1}H NMR spectrum of 1 (23 mM) in the presence of A (46 mM)) in methanol-d_4 at 298 K. Signals are assigned according to the resonance labels shown in Figure S4, with those shown in Black corresponding to 1, and those in Red to 2_A.

The structure of **1**, and its NMR resonances are shown in Table S7 and Figure S4. Resonances in dichloromethane-*d*2 at 243 K are also provided. The structure of the substitution product, **2A** is shown in Figure S5, and its NMR resonances are given in Table S8.

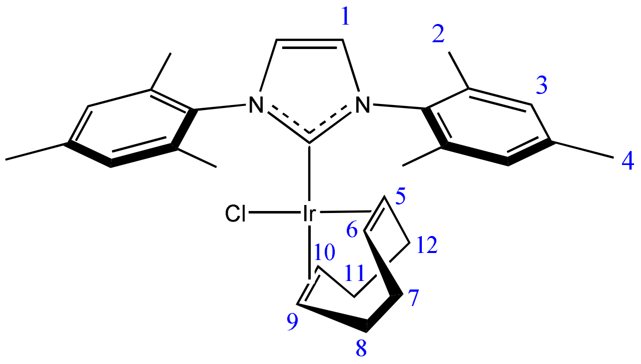

Figure S4: Structure of the SABRE precatalyst 1.

Table S7: ^{1}H NMR resonances of 1. The resonance labels corresponds to those shown in Figure S4

**Resonance Number**	**^{1}H / ppm**	
	**Methanol-*d*4, 298 K**	**Dichloromethane-*d*2, 243 K**
1	7.25	7.02
2	2.31	2.21/2.36/2.41
3	7.05	7.02
4	2.38	2.21/2.36/2.41 (now two signals as at low temperature N-C bond rotation in the IMes is slow)
5, 6 (trans to Cl)	3.10	3.04
7, 12	1.28, 1.65	1.0-1.6
8, 11	1.34, 1.72	1.0-1.6
9, 10 (cis to Cl)	4.02	4.07

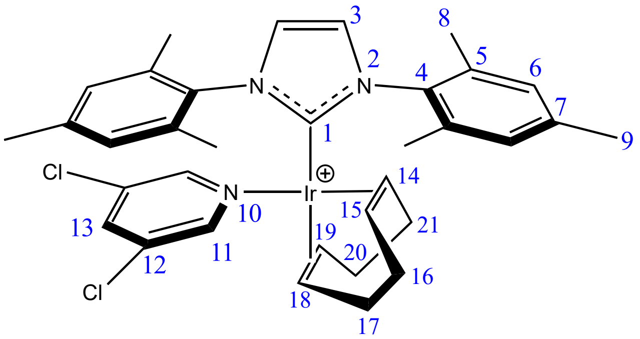

Figure S5: Structure of the subsitution product, 2_A.

Table S8: NMR resonances of 2_A in methanol-d_4 at 298 K. The resonance labels corresponds to those shown in Figure S5

Resonance Number	^1H / ppm	^{13}C / ppm	^{15}N / ppm
**1**		172.20	
**2**			195.80
**3**	7.40	125.50	
**4**		135.26	
**5**		138.48	
**6**	7.16	129.32	
**7**		140.22	
**8**	2.20	19.72	
**9**	2.46	19.90	
**10**			242.70
**11**	7.87, d, 2 Hz	147.60	
**12**			
**13**	8.19, t, 2 Hz	137.60	
**14, 15**	3.36	66.00	
**16, 21**	1.94, 2.14	-	
**17, 20**	1.70, 2.08	-	
**18, 19**	3.79	82.70	

The ratio between **1** and **2** at equilibirum was measured on separate samples when **A** or **B** was present as an ~13 fold excess relative to **1** in methanol-d_4 at a range of temperatures from 248 K to 303 K. The resulting equilibirium constant, K for these processes was determined by integration of the distinctive COD signals for each (appearing at δ 4.02 and δ 3.10 in **1**, but moving to δ 3.36 and δ 3.79 in $\mathbf{2_A}$) which were linked to their concentration. A Van't Hoff plot from these data (Figure S6) was used to determine $\Delta H^{\ominus}$ and $\Delta S^{\ominus}$, from which $\Delta G^{\ominus}$ could be calculated at a given temperature.

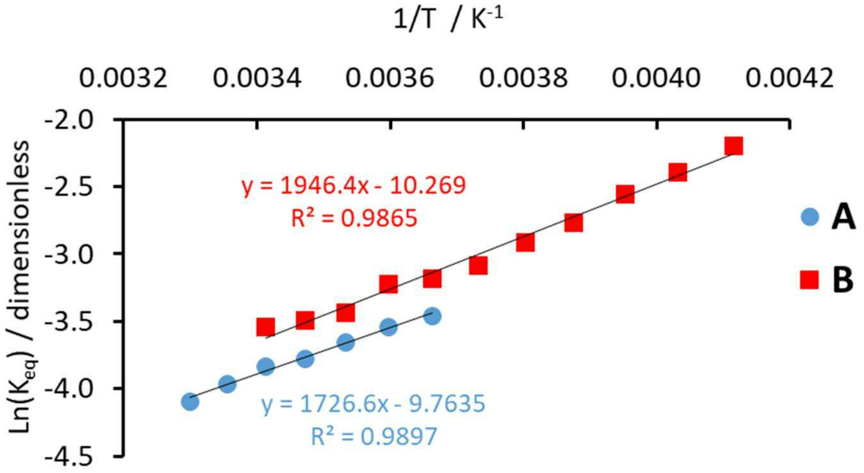

Figure S6: Van't Hoff plot allowing $\Delta H^{\ominus}$ and $\Delta S^{\ominus}$ for the equilbirum between 1 (5 mM) and 2_A (65 mM) in methanol-d_4 to be calculated.

<u>S3.2: Hydrogen addition to equilibrium mixtures of 1 and 2_A</u>

A solution of **1** (5 mM) and **2**$_A$ (50 mM) was cooled to 243 K and the reaction with pH$_2$ monitored by ^{1}H NMR spectroscopy. Some example NMR spectra are shown in Figures S7 and S8.

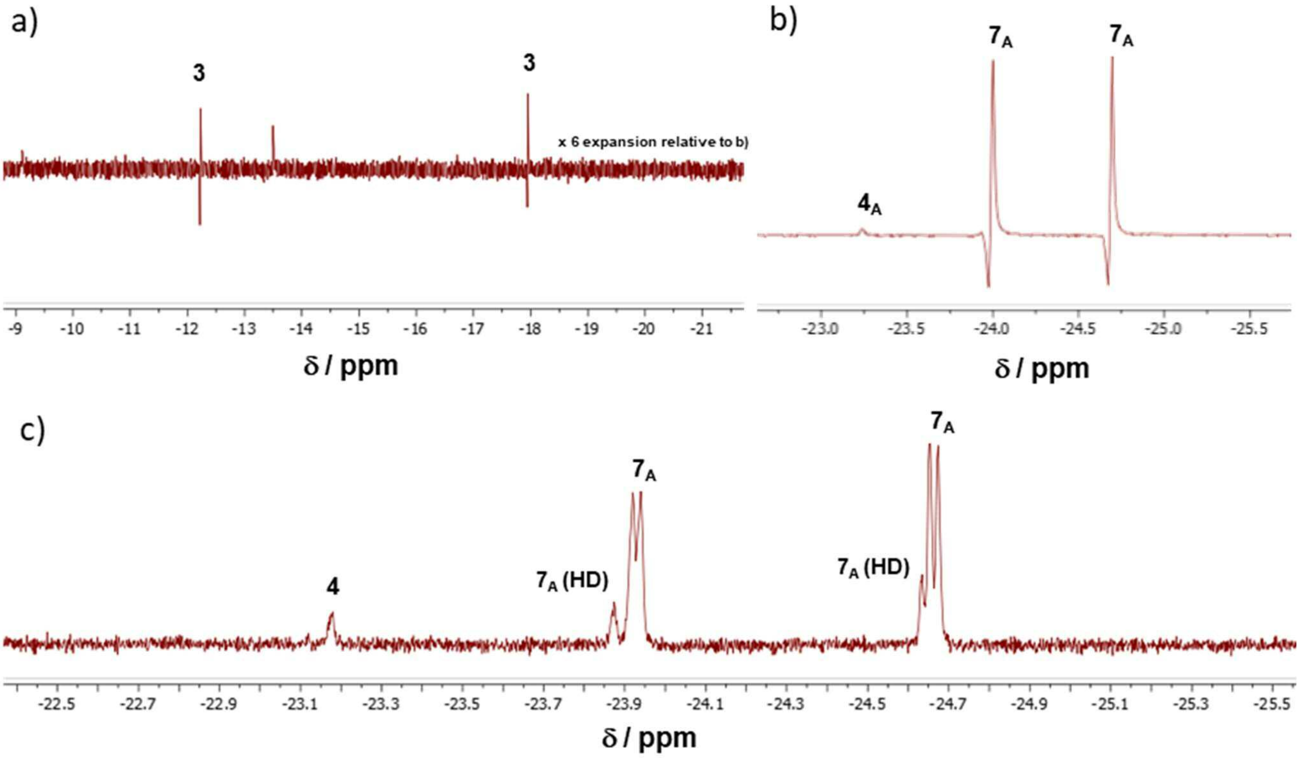

***Figure S7:** a)-b) Hyperpolarised ^{1}H NMR spectra recorded after addition of pH$_2$ to an equilibrium mixture of 1 (5 mM) and 2$_A$ (50 mM) in methanol-d$_4$ at 243 K. The resonances are labelled according to Figure 2 of the main paper. c) A thermally polarised spectrum of the same sample (not to scale).*

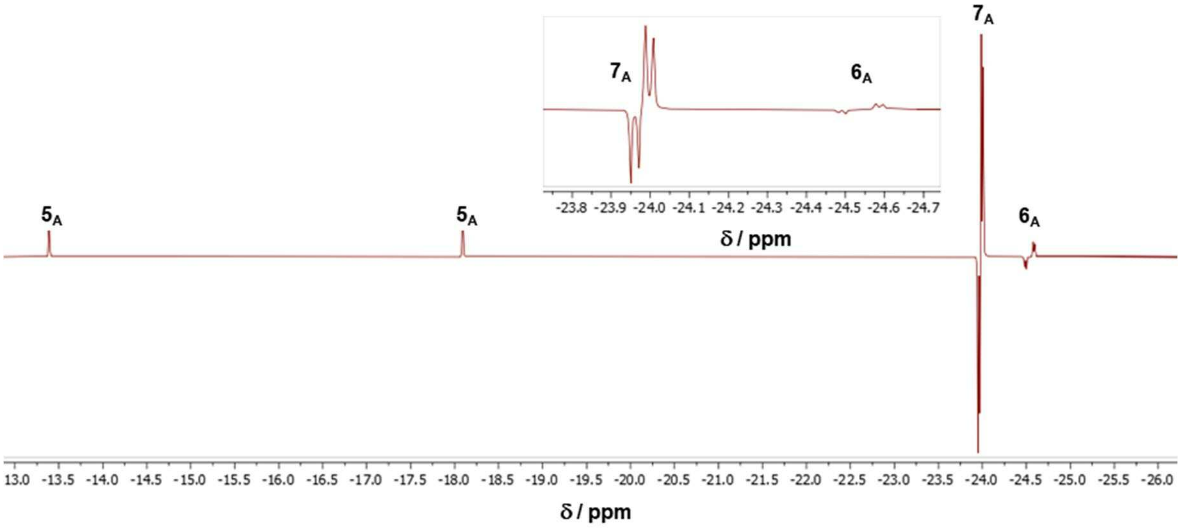

***Figure S8:** Hyperpolarised ^{1}H NMR spectra recorded after addition of pH$_2$ to an equilibrium mixture of 1 (5 mM) and 2$_A$ (50 mM) in dichloromethane-d$_2$ at 243 K. The resonances are labelled according to Figure 2 of the main paper. The inset shows an expansion of the region at ca −24 ppm.*

S3.3: Characterisation of 4_A

Solutions containing **3**, **4_A**, **5**, **6_A** and **7_A** were formed from hydrogen addition (3 bar) to an equilibrium mixture of **1** and **2_A**.

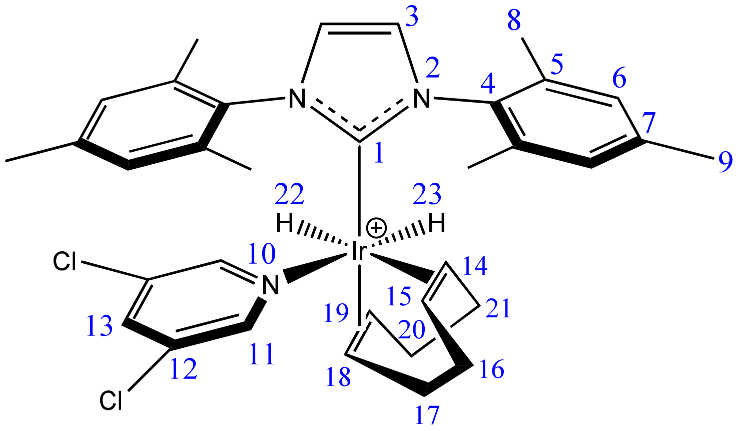

Figure S9: Structure of 4_A

Table S9: NMR resonances of 4_A in Methanol-d_4 at 243 K. The resonance labels correspond to those shown in Figure S9

Resonance Number	^{1}H / ppm	^{13}C / ppm	^{15}N / ppm
**1**		154.71	
**2**			197.4
**3**	7.42	124.73	
**4**		136.54	
**5**		135.18, 135.06	
**6**	7.13, 7.00	129.20, 129.15	
**7**		140.06	
**8**	2.08, 2.034	17.14, 16.87	
**9**	2.41	20.12	
**10**			232.2
**11**	8.44 *br*		
**12**			
**13**	8.35, *t J* = 2 Hz	137.43	
**14**	4.76	94.3	
**15**	4.35	89.1	
**16**	1.97, 2.08	30.91	
**17**	2.41, 1.83	34.47	
**18**	3.75	84.5	
**19**	5.09	82.8	
**20**	2.07, 1.88	25.29	
**21**	1.33, 2.30	29.90	
**22**	−12.0		
**23**	−17.85		

S3.4: Characterisation of 3

The low abundance of **3** necessitated characterisation in CD_2Cl_2 where higher solubility of **1** is beneficial. The first experiment used to characterise **3** was a 1D NOESY experiment which selectively saturated the hydride signal at δ −13.43 with a mixing time of 0.8 s. This revealed nOe interactions to a hydride resonance (δ −18.04) and resonances in the alkene region (δ 4.62 and δ 3.87) which suggests that COD is still bound. Another 1D NOESY was then used to selectively saturate the other hydride resonance (δ −18.04), using the same acquisition parameters as above, which revealed an nOe to three resonances in the alkene region (δ 4.62, δ 3.87 and δ 4.48) – further supporting a COD bound complex. A COSY spectrum revealed the fourth alkene resonance at δ 3.66 *via* coupling to that at δ 4.48. Each alkene resonance coupled to two different alkyl resonances in a COSY spectrum; used to assign all resonances within the bound COD ligand. The 1D NOESY spectra collected for both hydrides also revealed an nOe interaction to two alkyl signals (δ 2.15 and δ 2.27). These alkyl resonances appear as singlets of relative integral 3 in the ^{1}H NMR spectrum, and so are attributed to inequivalent *ortho* methyl proton in IMes. A weaker nOe interaction from these hydrides to δ 6.97 was also seen, thus assigning the *meta* aromatic protons. Furthermore, the nOe of both hydrides to the alkene resonance at δ 4.62 and δ 3.87 demonstrated that these alkene proton sites are *cis* to both hydrides, whereas the alkenes sites at δ 3.66 and δ 4.48 are *trans* to the hydride at δ −13.43, but *cis* to the hydride at δ −18.04.

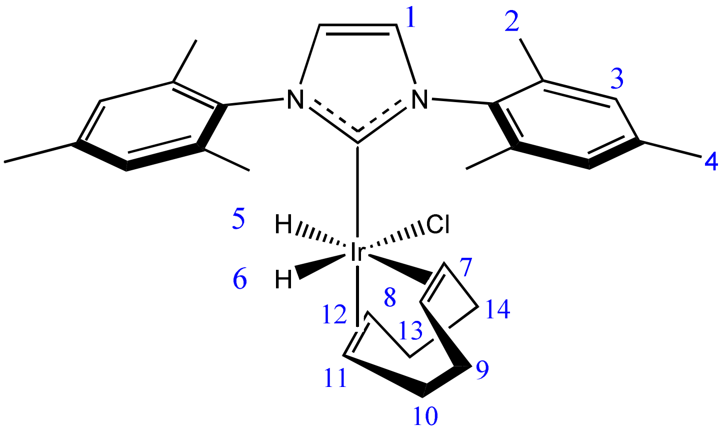

Figure S10: Structure of 3

Table S10: NMR resonances of 3 in dichloromethane-d_2 at 243 K. The resonance labels correspond to those shown in Figure S10

Resonance Number	^{1}H / ppm	^{13}C / ppm
**1**	6.91	122.93
**2**	2.15, 2.27	18.67
**3**	6.97	128.55
**4**	2.35	
**5**	−13.43	
**6**	−18.04	
**7**	3.66	92.0
**8**	4.48	85.70
**9**	1.52, 1.84	
**10**	2.07, 2.64	
**11**	4.62	73. 6
**12**	3.87	79.95
**13**	1.68, 2.25	
**14**	1.97, 2.47	

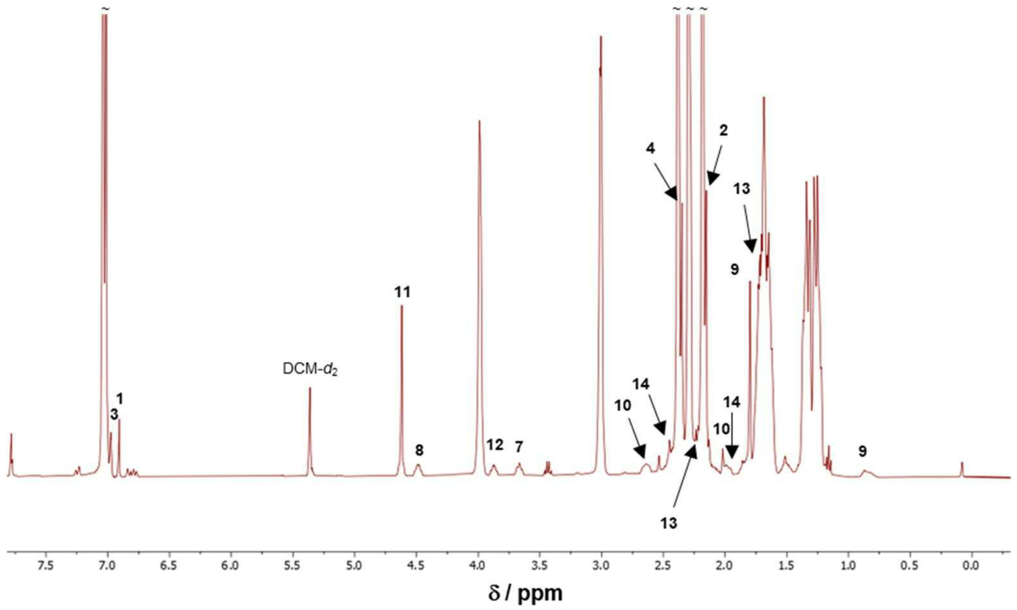

Figure S11: ^{1}H NMR spectra showing the resonances for 3 recorded after addition of H_2 to an equilibrium mixture of 1 (5 mM) and 2_A (50 mM) in dichloromethane-d_2 at 243 K. Note the resonances for 3, labelled according to Figure S10) are weak in comparison to those of 2_A (not assigned).

S3.5: Characterisation of 6_A

A 1D NOESY experiment was used to selectively saturate the hydride resonance of $\mathbf{6_A}$ at δ −23.87 with a mixing time of 0.3 s. An nOe interaction was observed to the resonance at δ 8.82, a signal which has a doublet multiplicity and a relative integral of *ca* 2, suggesting that this resonance belonged to the *ortho* protons of a bound 3,5-dichloropyridine. A 1D NOESY experiment exciting the other hydride signal (δ - 24.10) revealed nOe interactions to δ 8.82 and δ 8.25, both with doublet multiplicity and a relative integral of *ca* 2, indicative of the *ortho* protons of bound 3,5-dichloropyridine. This suggests that both hydrides are *cis* to a 3,5-dichloropyridine ligand (δ 8.82) and the hydride at δ −24.10 is *cis* to a second 3,5-dichloropyridine ligand, with the hydride (δ −23.87) *trans* to bound **A**. Both hydrides displayed an nOe to alkyl signals at δ 2.16 and δ 2.21, which both had a relative integral of *ca* 3 and were assigned to the *ortho* methyl protons of IMes. The relative positions of these signals were determined by the strength of the nOe interaction between the hydride and the methyl resonance. Furthermore, the hydride signals also showed an nOe to a resonance at δ 6.79, a singlet with a relative integral of *ca* 2 in a ^{1}H NMR spectrum, thus this was attributed to the *meta* aromatic protons of IMes. Now that the *meta* aromatic protons of IMes have been assigned (δ 6.79), the resonance for the imidazole protons can now be assigned. A resonance at δ 6.82 is a singlet, with a relative integral of *ca* 2, with a HMQC revealing it directly bound to an alkene carbon. Thus, δ 6.82 can be assigned to the imidazole proton of IMes.

^{15}N NMR spectra were recorded to further support two bound 3,5-dichloropyridine ligands (δ 270.00 and δ 247.83) in the complex.

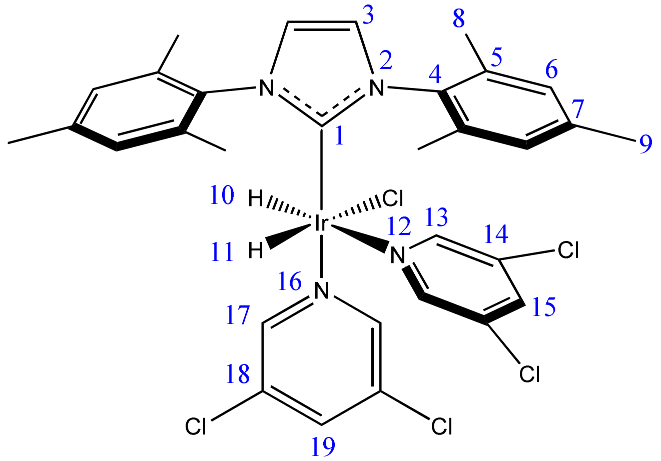

Figure S12: Structure of 6_A, its NMR resonances are given in Table S11.

Table S11: NMR resonances of 6_A. The resonance labels correspond to those shown in Figure S12.

	Dichloromethane-d_2 at 243 K			Methanol-d_4 at 247 K		
Resonance Number	^1H / ppm	^{13}C / ppm	^{15}N / ppm	^1H / ppm	^{13}C / ppm	^{15}N / ppm
1		150.38			149.93	
2			192.80			193.33
3	6.82	121.90		7.06	122.03	
4		138.10			138.21	
5		135.90			135.80, 135.82	
6	6.79	128.33		6.86, 6.78	128.21, 128.29	
7		138.08			138.15	
8	2.16, 2.21	-		2.21	17.50, 17.77	
9	2.24	-		2.25	19.92	
10	−23.87			−23.86		
11	−24.10			−24.64		
12			270.00			--
13	8.25, d	147.25		8.42, br	--	
14		131.96			--	
15	7.49, t	133.71		7.94, t 2Hz	134.18	
16			247.83			248.38
17	8.82, d	152.75		8.73, d 2Hz	152.25	
18		132.27			132.20	
19	7.55, t	135.11		7.95, t 2Hz	135.51	

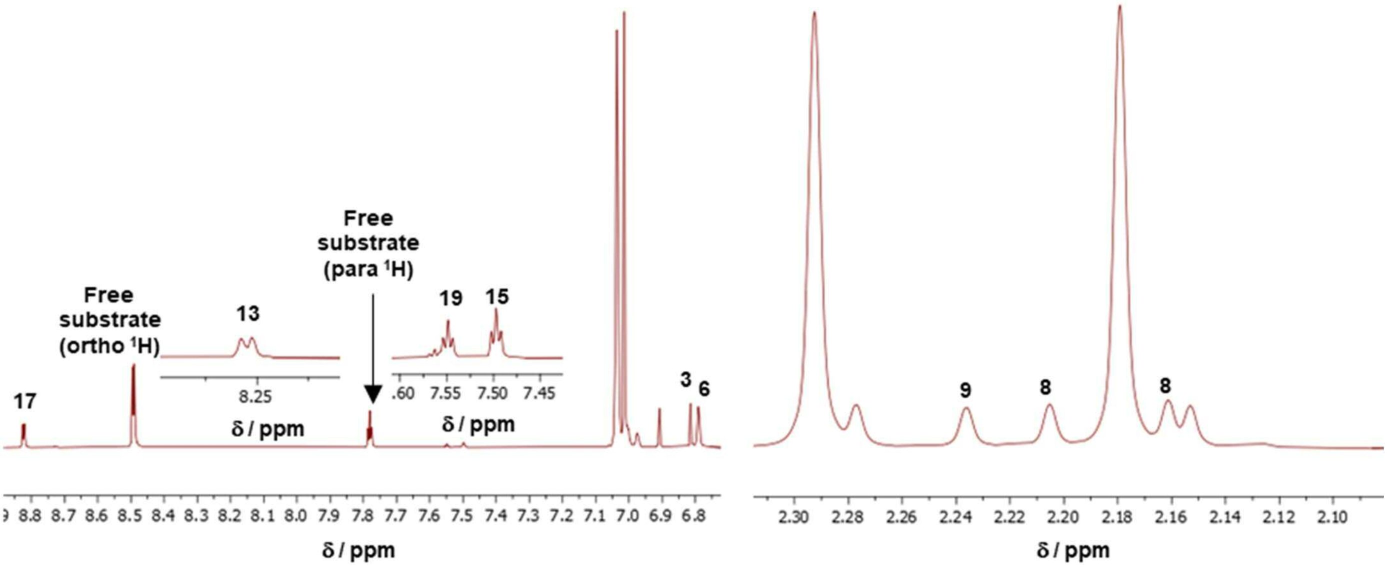

Figure S13: ^1H NMR spectra showing the resonances for 6_A recorded after addition of H_2 to an equilibrium mixture of 1 (5 mM) and 2_A (50 mM) in dichloromethane-d_2 at 243 K. Note the resonances for 6_A labelled according to Figure S12, are weak in comparison to those of 2_A (not assigned).

A series of ^{1}H NMR spectrea of the hydride ligands of **6A** in dichloromethane-d_2, recorded at different temperatures are shown in Figure S14, with the corresponding *J* coupling values in Table S12.

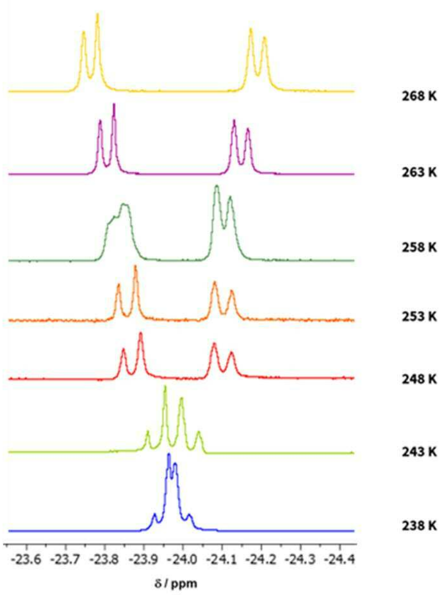

Figure S14: ^{1}H NMR spectra of 6_A between 238 K and 268 K in dichloromethane-d_2 which show the temperature dependence of the hydride chemical shifts, resulting in the AX spin-system changing to AB upon cooling. Associated J coupling values are presented in Table S12.

Table S12: The difference in chemical shift (D), coupling constant (J_{HH}) and AX/AB spin-system assignment for the hydride signals of 6_A between 238 K and 268 K in dichloromethane-d_2.

Temperature / K	J_{HH} / Hz	D / Hz	D/J_{HH} / dimensionless	Spin System
238	7.97	13.4	1.68	AB
243	7.97	15.4	1.93	AB
248	7.97	41.7	5.23	AB
253	8.26	43.8	5.30	AB
258	8.28	61.1	7.37	AB
263	8.09	77.2	9.55	AB
268	7.98	96.7	12.1	AX

S3.6: X-Ray Crystallography of 6_A

6_A was prepared in methanol-d_4 (0.6 mL) by reaction of [IrCl(η^2-η^2-COD)(IMes)] (**1**) (5 mM) and **A** (50 mM) with 3 bar H_2 and left at room temperature for 6 hours. At this point it was cooled to 278 K in a fridge and left for several weeks. Single crystals formed. A suitable crystal was selected and mounted on an Oxford-Diffraction SuperNova dual-source X-ray diffractometer equipped with copper and molybdenum sources and a HyPix-6000HE detector. Cooling to 110 K was achieved using an Oxford Instruments Cryojet. Using Olex2, the structure was solved with the SHELXT structure solution program using Intrinsic Phasing and refined with the SHELXL refinement package using Least Squares minimisation. Crystallography details are given in Table S13. The structure of **6_A** is shown in the main paper, Figure 2a.

Table S13: X-Ray Crystallography details for 6_A

Empirical formula	$C_{31}H_{32}Cl_5IrN_4$
Formula weight/ Da	830.05
Temperature/K	110.00(10)
Crystal system	monoclinic
Space group	$P2_1/c$
a/Å	12.08687(9)
b/Å	24.32050(13)
c/Å	11.89130(10)
α/°	90
β/°	114.4845(10)
γ/°	90
Volume/Å^3	3181.21(5)
Z	4
ρ_{calc}g/cm^3	1.733
μ/mm^{-1}	12.209
F(000)	1632.0
Crystal size/mm^3	0.116 × 0.101 × 0.069
Radiation	Cu Kα (λ = 1.54184)
2Θ range for data collection/°	7.27 to 134.16
Index ranges	$-14 \le h \le 14$, $-12 \le k \le 29$, $-14 \le l \le 14$
Reflections collected	29221
Independent reflections	5680 [R_{int} = 0.0259, R_{sigma} = 0.0176]
Data/restraints/parameters	5680/0/385
Goodness-of-fit on F^2	1.080
Final R indexes [I>=2σ (I)]	R_1 = 0.0187, wR_2 = 0.0430
Final R indexes [all data]	R_1 = 0.0192, wR_2 = 0.0433
Largest diff. peak/hole / e Å^{-3}	1.95/-0.45
Ir-C_1 bond distance /Å	1.985
Ir-Cl bond distance /Å	2.483
Ir-N_{12} bond distance /Å	2.238
Ir-N_{16} bond distance /Å	2.137
Ir-H_{10} bond distance /Å	1.483
Ir-H_{11} bond distance /Å	1.514

S3.7: Characterisation of $\mathbf{2_B}$

An NMR sample containing [IrCl(η^2- η^2-COD)(IMes)] (**1**) (5 mM) and **B** (25 mM) in a ~ 1 : 5 ratio was prepared in methanol-d_4 (0.6 mL) and a series of NMR experiments recorded at 243 K. This allowed [Ir(**B**)(η^2- η^2-COD)(IMes)]Cl, $\mathbf{2_B}$, to be characterised using 2D NMR. Its structure is shown in Figure S15 and its NMR resonances are detailed in Table S14. Notably, the ratio of free **B** to $\mathbf{2_B}$ was 4.23 : 0.48, with 1 equivalent of **1** remaining.

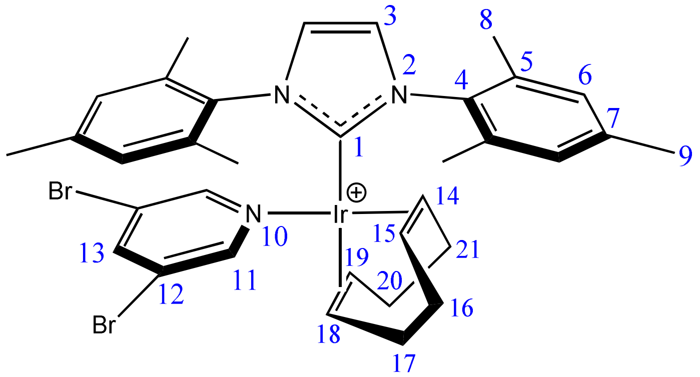

Figure S15: Structure of 2_B, its NMR resonances are given in Table S14.

Table S14: NMR resonances of 2_B in methanold-d_4 at 243 K. The resonance labels correspond to those shown in Figure S15.

Resonance Number	^{1}H / ppm	^{13}C / ppm	^{15}N / ppm
**1**		171.92	
**2**			195.88
**3**	7.49	125.48	
**4**		136.19	
**5**		138.72, 137.29	
**6**	7.25 *br*		
**7**		140.09	
**8**	2.22	17.29	
**9**	2.48	20.20	
**10**			252.15
**11**	7.49, *t* 2 Hz	149.82	
**12**		121.79	
**13**	8.02, *t* 2 Hz	142.97	
**14, 15**	3.36	66.00	
**16, 21**	2.08, 1.71	28.83	
**17, 20**	1.93, 1.69	32.09	
**18, 19**	3.75	82.53	

S3.8: Characterisation of 4_B

The sample described in S3.7 was then cooled in a dry ice acetone bath and 3 bar of *p*H_2 was added to the NMR tube before being reintroduced into the NMR spectrometer (at 243 K). The sample took several minutes to reach 243 K. During this period, PHIP-enhanced hydride signals were also detected at −12.02 and −17.92, and −13.33 and −18.50 in agreement with the detection of $[Ir(H)_2(\mathbf{B})(\eta^2\text{-}\eta^2\text{-COD})(IMes)]Cl$, **$4_B$** and $[Ir(Cl)(H)_2(\eta^2\text{-}\eta^2\text{-COD})(IMes)]$, **3**. **$4_B$** was then characterised using thermally polarised 2D NMR methods. Its structure is shown in Figure S16 and its NMR resonances are detailed in Table S15. The characterisation of **3** was detailed in Section S3.4

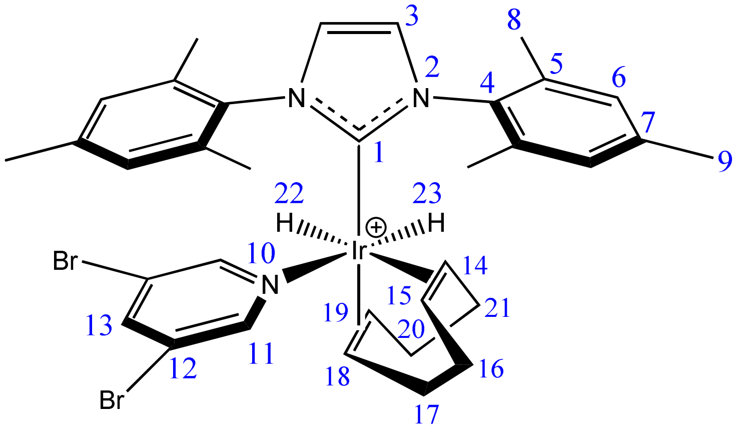

Figure S16 Structure of 4_B, its NMR resonances are given in Table S15.

Table S15: NMR resonances of 4_B in methanold-d_4 at 243 K. The resonance labels correspond to those shown in Figure S16.

**Resonance Number**	**^{1}H / ppm**	**^{13}C / ppm**	**^{15}N / ppm**
**1**		153.54	
**2**			197.3
**3**	7.42	124.70	
**4**		136.52	
**5**		135.12, 135.08	
**6**	7.13, 7.03	129.20, 129.19	
**7**		140.04	
**8**	2.06, 2.02	17.07, 16.91	
**9**	2.42	20.20	
**10**			-
**11**	8.55 *br*	-	
**12**		122.05	
**13**	8.61, *t*, 2 Hz	142.82	
**14**	4.74	93.28	
**15**	4.30	89.65	
**16**	2.05, 2.07	31.13	
**17**	2.40, 1.84	34.23	
**18**	5.10	82.84	
**19**	3.74	85.84	
**20**	2.04, 1.93	25.44	
**21**	1.83, 2.29	29.95	
**22**	−12.02		
**23**	−17.89		

S3.9: Characterisation of 6_B

Upon warming the solution described in S2.8, **6_B** forms and was characterised at low temperature. Its structure is shown in Figure S17 and its NMR resonances are given in Table S16.

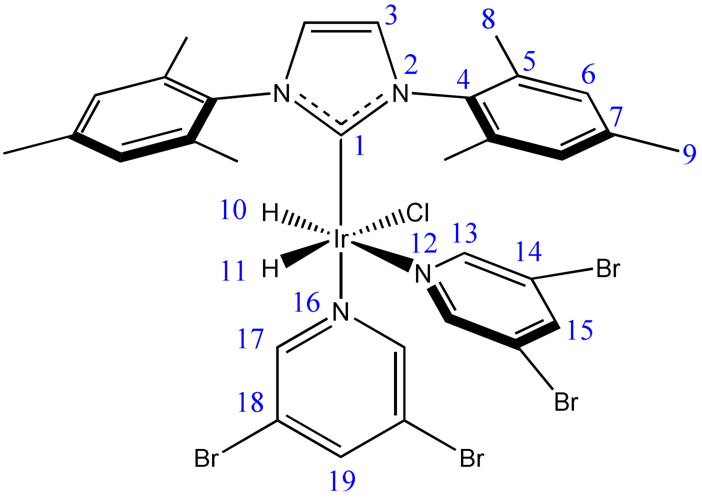

Figure S17: Structure of 6_B, its NMR resonances are given in Table S16.

Table S16: NMR resonances of 6_B at 243 K. The resonance labels correspond to those shown in Figure S17.

	**Methanol-*d*$_4$, 243 K**			***Dichloromethane-d*$_2$, 243 K**		
**Resonance Number**	**^{1}H / ppm**	**^{13}C / ppm**	**^{15}N / ppm**	**^{1}H / ppm**	**^{13}C / ppm**	**^{15}N / ppm**
**1**		149.6			150.24	
**2**			193.29			192.57
**3**	7.06	122.00		6.81	121.77	
**4**		138.11			138.10	
**5**		135.40, 136.07			136.00, 135.92	
**6**	6.87, 6.79	128.22, 128.28		6.80, br	128.42	
**7**		137.89			138.06	
**8**	2.17 *br*	17.49, 17.77		2.21, 2.16	18.28, 18.59	
**9**	2.25	20.00		2.24	21.10	
**10**	−23.82, *d J* = 8.3 Hz			−23.97, *d J* = 8.2 Hz		
**11**	−24.64, *d J* = 8.3 Hz			−24.03, *d J* = 8.2 Hz		
**12**			-			-
**13**	9.17 br			9.34 br, 8.52 br	149.56, 156.38	
**14**		120.78			120.40	
**15**	8.21, t	140.79		7.77 t 1.9 Hz	138.79	
**16**			298.5			249.74
**17**	8.82, d, 1.9 Hz	154.44		8.95 d 1.9 Hz	154.96	
**18**		120.07			120.43	
**19**	8.20, t 1.9 Hz	139.33		7.83 t 1.9 Hz	140.35	

S3.10: X-Ray Crystallography of 6_B

6_B was prepared in methanol-*d*$_4$ (0.6 mL) by reaction of [IrCl(η^2-η^2-COD)(IMes)] (**1**) (5 mM) and **A** (50 mM) with 3 bar H_2 and left at room temperature for 6 hours. At this point it was cooled to 278 K in a fridge and left for several weeks. Single crystals formed. A suitable crystal was selected and mounted on an Oxford-Diffraction SuperNova dual-source X-ray diffractometer equipped with copper and molybdenum sources and a HyPix-6000HE detector. Cooling to 110 K was achieved using an Oxford Instruments Cryojet. Using Olex2, the structure was solved with the SHELXT structure solution program using Intrinsic Phasing and refined with the SHELXL refinement package using Least Squares minimisation.
The crystal gave relatively streaked reflections with evidence of twinning. This is believed to be the cause of high residual density peaks centred around the iridium. These results represent the best absorption corrections we were able to perform. Attempts to solve the crystal as a multi-component twin gave R1 and wR2 which were significantly poorer (5.79 and 18.98%, respectively) than modelling using a single component, although the peak and trough residual densities were improved, 2.3 and -2.1. Ir-H bond lengths were constrained to be 1.77 angstroms as allowing them to refine led to unfeasibly short Ir-H bond lengths. Crystallography details are given in Table S17. The structure of **6_B** is shown in Figure S18.

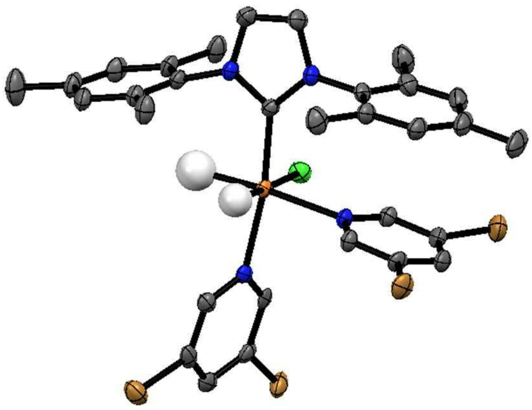

Figure S18: X-Ray Crystallography structure of 6_B with thermal ellipsoids shown at 50% probability. Note that any solvent of crystallisation, and all non-hydride H atoms have been omitted for clarity. Grey corresponds to carbon, blue to nitrogen, brown to bromine, orange to iridium, green to chlorine, and white to hydrogen.

Table S17: X-Ray Crystallography details for 6_B

Empirical formula	$C_{31}H_{32}N_4ClBr_4Ir$
Formula weight/ Da	1007.89
Temperature/K	110.00(10)
Crystal system	monoclinic
Space group	$P2_1/c$
a/Å	11.91172(19)
b/Å	24.7542(3)
c/Å	12.4277(2)
α/°	90
β/°	115.347(2)
γ/°	90
Volume/Å^3	3311.73(10)
Z	4
ρ_{calc}g/cm^3	2.021
μ/mm^{-1}	14.414
F(000)	1920.0
Crystal size/mm^3	0.118 × 0.069 × 0.026
Radiation	Cu Kα (λ = 1.54184)
2Θ range for data collection/°	8.214 to 147.502
Index ranges	-14 ≤ h ≤ 14, -29 ≤ k ≤ 27, -14 ≤ l ≤ 14
Reflections collected	36341
Independent reflections	5904 [R_{int} = 0.0656, R_{sigma} = 0.0378]
Data/restraints/parameters	5904/1/378
Goodness-of-fit on F^2	1.033
Final R indexes [I>=2σ (I)]	R_1 = 0.0459, wR_2 = 0.1189
Final R indexes [all data]	R_1 = 0.0489, wR_2 = 0.1209
Largest diff. peak/hole / e Å^{-3}	3.27/-1.35
Ir-C_1 bond distance /Å	2.008
Ir-Cl bond distance /Å	2.550
Ir-N_{12} bond distance /Å	2.148
Ir-N_{16} bond distance /Å	2.163
Ir-H_{10} bond distance /Å	1.770 (fixed)
Ir-H_{11} bond distance /Å	1.770 (fixed)

S4: SABRE Hyperpolarisation of A and B in the presence of a sulfoxide coligand

Samples containing [IrCl(η²-η²-COD)(IMes) (**1**) (5 mM) and the substrate 3,5-dichloropyridine (**A**) or 3,5-dibromoopyridine (**B**) (50 mM) with either dimethylsulfoxde or diphenylsulfoxide (25 mM) were dissolved in either methanol-*d*₄ or dichloromethane-*d*₂ (0.6 mL) and exposed to 3-bar H_2 gas for 2-3 hours to form SABRE-active iridium dihydride complexes. After this point, the H_2 atmosphere was replaced with *p*H_2 and a series of hyperpolarisation measurements were performed by shaking the sample for 10 seconds in the stray field of a 9.4 T spectrometer (*ca* 6.5 mT). NMR spectral acquisition commenced immediately after the sample was dropped into the spectrometer, and the hyperpolarisation process was repeated multiple times for each sample by replacing the spent *p*H_2 with fresh *p*H_2 before re-shaking the solution as described.

^{1}H NMR signal enhancements for **A** and **B** are given in Tables S18 and S19 respectively. ^{1}H T_1 relaxation times are also given. ^{13}C and ^{15}N NMR signal enhancements for **A** and **B** are given in Tables S20 and S21 respectively.

Table S18: ^{1}H NMR signal enhancements and T_1 relaxation times for A in the presence of a sulfoxide coligand.

		DMSO				DPSO	
Solvent	Site	*Ortho* bound	*Ortho* free	*Para* free	*Para* bound	*Ortho* free	*Para* free
Methanol-d_4	^{1}H E/ fold	785 ± 36	1119 ± 14	870 ± 28	1062 ± 104	290 ± 16	457 ± 10
Dichloromethane-d_2	^{1}H E/ fold	6604 ± 452	4350 ± 65	4413 ± 22	8633 ± 276	1287 ± 37	259 ± 26

Table S19: ^{1}H NMR signal enhancements and T_1 relaxation times for B in the presence of a sulfoxide coligand.

		DMSO				DPSO	
Solvent	Site	*Ortho* bound	*Ortho* free	*Para* free	*Para* bound	*Ortho* free	*Para* free
Methanol-d_4	^{1}H E/ fold	1023 ± 103	1172 ± 42	1088 ± 66	3310 ± 165	504 ± 12	348 ± 9
Dichloromethane-d_2	^{1}H E/ fold	1639 ± 57	1355 ± 21	1613 ± 46	8274 ± 170	912 ± 129	617 ± 230

***Table S20:** ^{13}C NMR signal enhancements for A and B with the indicated sulfoxide co-ligands. The NMR signal enhancements are recorded by shaking a sample of [IrCl(η^2-η^2-COD)(IMes)] and A or B (50 mM) with either DMSO or DPSO (25 mM) in the indicated solvent with 3-bar pH_2 for 10 seconds in a mu metal shield at 1 mG.*

	**A** 50 mM		**B** 50 mM	
	DMSO	DPSO	DMSO	DPSO
Methanol-d_4	IMes carbene: 178 ± 17<br>o free: 356 ± 41<br>o bound: 344 ± 42<br>p free: 84 ± 9<br>p bound: 119 ± 14<br>m free: 1221 ± 70<br>m bound: 148 ± 5	IMes carbene: 43 ± 7<br>DPSO: 74 ± 6<br>o free: 159 ± 26<br>o bound: 170 ± 5<br>p free: 152 ± 3<br>p bound: 62 ± 4<br>m free: 349 ± 12<br>m bound: 29 ± 1	IMes carbene: 144 ± 6<br>o free: 362 ± 15<br>o bound: 331 ± 20<br>p free: 96 ± 11<br>p bound: 168 ± 11<br>m free: 1553 ± 59<br>m bound: 162 ± 5	IMes carbene: 14 ± 1<br>o free: 89 ± 2<br>o bound: 67 ± 10<br>p free: 17 ± 1<br>p bound: 39 ± 1<br>m free: 401 ± 8<br>m bound: 33 ± 1
Dichloromethane-d_2	IMes carbene: 119 ± 12<br>o free: 748 ± 61<br>o bound: 726 ± 50<br>p free: 246 ± 15<br>p bound: 306 ± 17<br>m free: 732 ± 67<br>m bound: 48 ± 9	IMes carbene: 81 ± 3<br>DPSO: 110 ± 9<br>o free: 288 ± 23<br>o bound: 385 ± 16<br>p free: 80 ± 1<br>p bound: 112 ± 12<br>m free: 625 ± 46<br>m bound: 54 ± 9	IMes carbene: 4 ± 1<br>o free: 316 ± 30<br>o bound: 410 ± 41<br>p free: 5 ± 1<br>p bound: 198 ± 20<br>m free: 1125 ± 52<br>m bound: 99 ± 4	IMes carbene: 3 ± 1<br>o free: 136 ± 13<br>o bound: 142 ± 27<br>p free: 2 ± 1<br>p bound: 85 ± 11<br>m free: 422 ± 7<br>m bound: 21 ±1

***Table S21:** ^{15}N NMR signal enhancements for A and B with the indicated sulfoxide co-ligands. The NMR signal enhancements are recorded by shaking a sample of [IrCl(η^2-η^2-COD)(IMes)] and A or B (50 mM) with either DMSO or DPSO (25 mM) in the indicated solvent with 3-bar pH_2 for 10 seconds in a mu metal shield at 6 mG.*

	**A** 50 mM		**B** 50 mM	
	DMSO	DPSO	DMSO	DPSO
Methanol-d_4	Free: 2606 ± 476<br>Bound: 269 ± 34<br>Carbene: 737 ± 126	Free: 3296 ± 44<br>Bound: 1257 ± 24	Free: 3840 ± 101<br>Bound: 1741 ± 14<br>Carbene: 932 ± 48	Free: 2681 ± 96
Dichloromethane-d_2	Free: 8086 ± 180<br>Bound: 331 ± 11	Free: 4216 ± 141<br>Bound: 339 ± 24<br>Carbene: 2615 ± 3	Free: 10,422 ± 632<br>Bound: 1,018 ± 69<br>Carbene: 287 ± 90	Free: 3858 ± 3<br>Bound: 260 ± 38

^{15}N NMR signal enhancements with DMSO were optimized further by variation of the polarization transfer field, substrate loading and shaking time. The effect of these variables is shown in Figure S19.

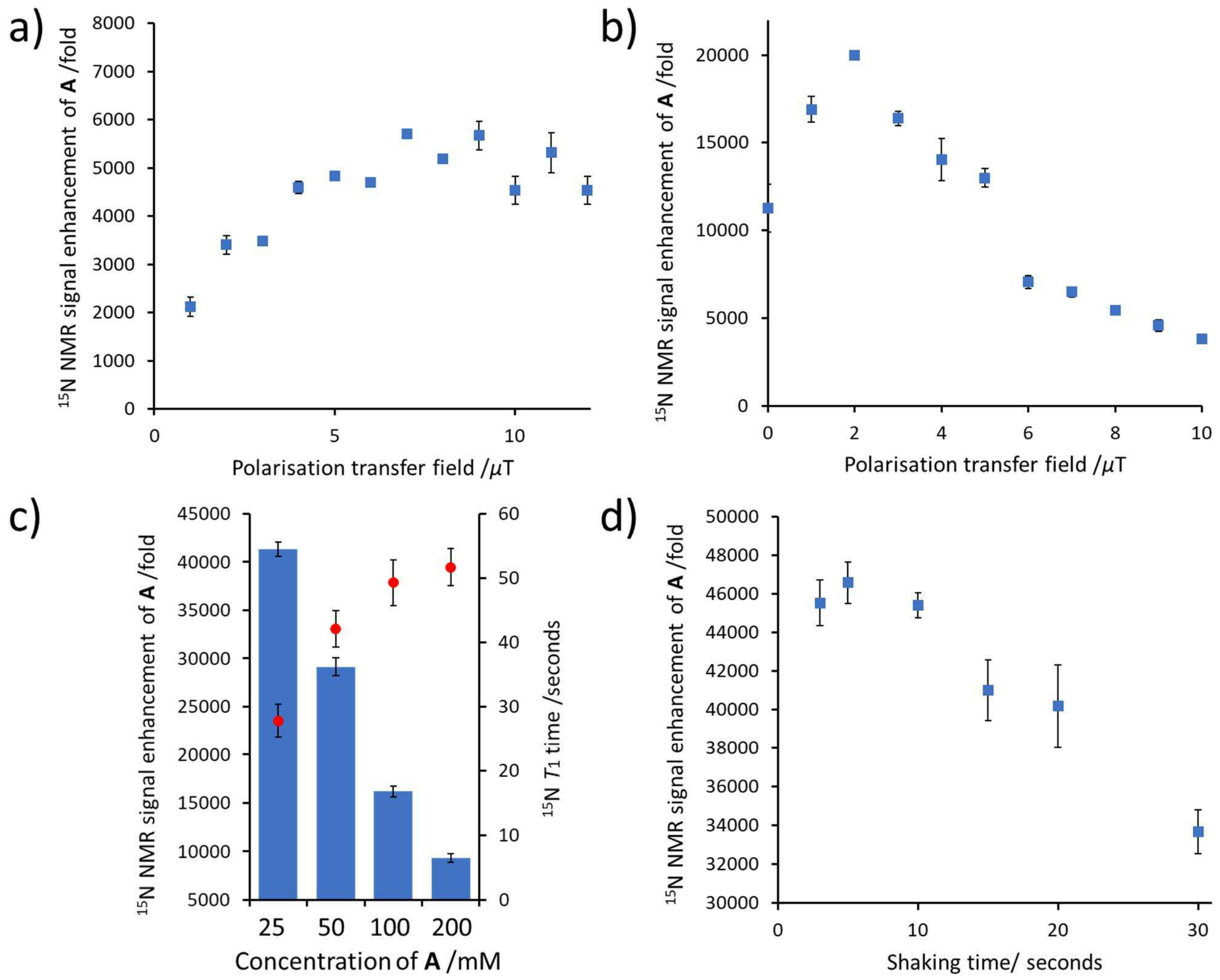

***Figure S19:** a) ^{15}N NMR signal enhancements for A (100 mM) as a function of polarisation transfer field recorded by shaking a sample of it with [IrCl(η^2-η^2-COD)(IMes)] (5 mM) in methanol-d_4 with 3-bar pH_2 for 10 seconds at the indicated transfer field b) ^{15}N NMR signal enhancements for A (100 mM) as a function of polarisation transfer field recorded by shaking a sample of it with [IrCl(η^2-η^2-COD)(IMes)] (5 mM) and DMSO (50 mM) in dichloromethane-d_2 with 3 bar pH_2 for 10 seconds at the indicated transfer field. c) ^{15}N NMR signal enhancements (left axis) and T_1 times (right axis) for A as a function of loading relative to 1 (5 mM) with DMSO (50 mM) in dichloromethane-d_2 at a 2 mG transfer field. d) ^{15}N NMR signal enhancements for A (25 mM) as a function of shaking time with 1 (5 mM) and DMSO (50 mM) in dichloromethane-d_2 at a 2 mG transfer field.*

^{15}N T_1 relaxation times for **A** (50 eq) with **1** (5 mM) and DMSO (25 mM) in dichloromethane-d_2 and tetrahydrofuran-d_8 (0.6 mL) with pH$_2$ (3 bar) were also measured at 7.1 T, 9.4 T and 11.7 T (Figure S20). These were recorded in the same way as described for ^{1}H in section S2, but with 14-19.5° flip angles and time spacings of 5-8 seconds depending on the magnetic field.

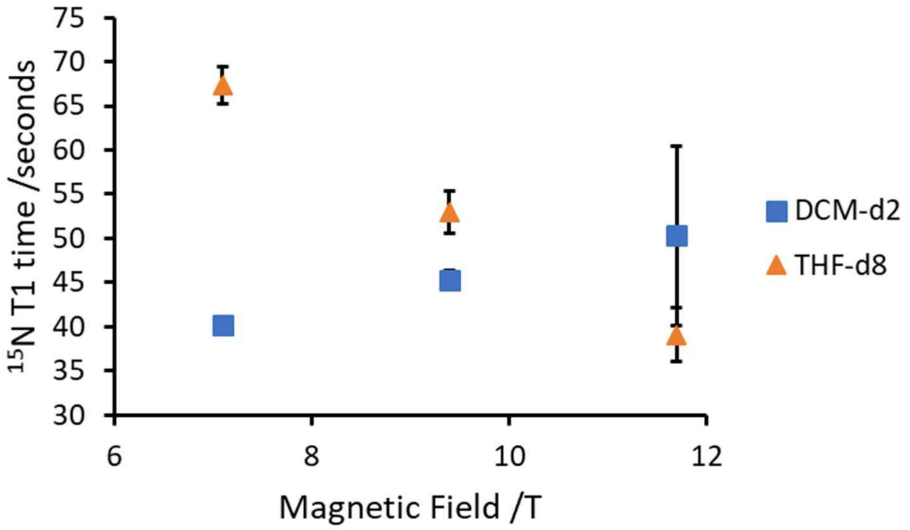

***Figure S20:** ^{15}N T_1 times for A (250 mM) with 1 (5 mM) and DMSO (25 mM) in dichloromethane-d_2 and tetrahydrofuran-d_8 (0.6 mL) with pH_2 (3 bar) as a function of magnetic field.*

S5: Characterisation of sulfoxide-containing metal complexes involved in SABRE

S5.1: 2D NMR characterisation of 8_A

8$_A$ was prepared by reaction of **1** (10 mM), **A** (25 mM) and DMSO (25 mM) in methanol-d_4 (0.6 mL) with H_2 (3 bar) for a few hours at room temperature before being cooled to 245 K and characterised using 2D NMR. NOE connections were found from both hydride signals to IMes resonances at δ 7.21, δ 6.85, δ 6.79, δ 2.27 and δ 2.16, and the bound sulfoxide resonances at δ 2.77 and δ 3.14. The relative integral intensities confirmed these were due to inequivalent CH_3 sites of the bound DMSO, rather than from two different ligated DMSO. Observation of their signals nOe to both hydries confirms that the IMes and DMSO are *trans* as no nOe peaks were observed between them. NOE from the hydride at δ −23.11 to the bound *ortho* signal of **A** at δ 8.76 confirmed the orientation of **A** *cis* to this hydride. ^{13}C NMR resonances were assigned using HMQC.

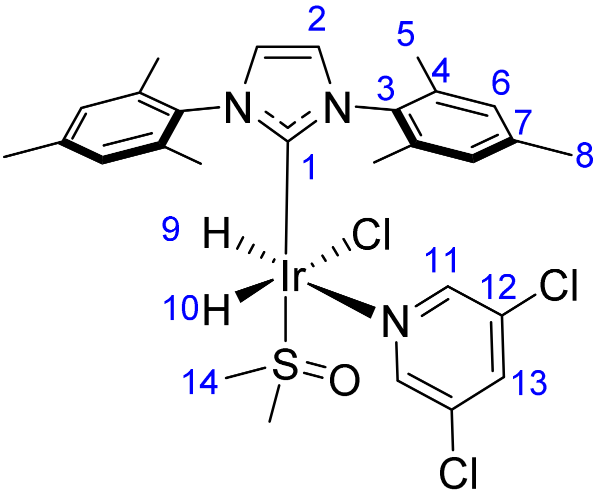

Figure S21: Structure of 8$_A$, its NMR resonances are given in Table S22.

Table S22: NMR resonances of 8$_A$. The resonance labels correspond to those shown in Figure S21.

	Methanol-d$_4$ at 245 K		*Dichloromethane-d$_2$ at 245 K**	
**Resonance Number**	**^{1}H / ppm**	**^{13}C / ppm**	**^{1}H / ppm**	**^{13}C / ppm**
**1**		158.81		159.61
**2**	7.21	122.90	6.92	122.47
**3**		137.64		137.55
**4**		135.29, 135.57		135.62, 135.77
**5**	2.16	17.33, 17.55	2.12, 2.15	18.14/18.43
**6**	6.79, 6.85	128.32, 128.25	6.78, 6.80	128.35, 128.39
**7**		138.48		138.58
**8**	2.27	19.96	2.26	21.04
**9**	−23.55 (*d*, 8.5 Hz)		−23.76 (*d*, 7.5 Hz)	
**10**	−23.11(*d*, 8.5 Hz)		−22.98 (*d*, 7.5 Hz)	
**11**	8.76	-	8.74	145.51
**12**		131.72		131.55
**13**	8.01	134.63	7.58	134.31
**14**	2.77, 3.14	42.27, 54.99	3.07	55.45

*^{15}N signals in dichloromethane at 194.70 (IMes) and 256.12 (bound **A**)

S5.2: 2D NMR characterisation of 8_B

8_B was prepared by the reaction of **1** (10 mM), **B** (25 mM) and DMSO (25 mM) in methanol-*d*$_4$ (0.6 mL) with H_2 (3 bar) over a few hours at room temperature, before being cooled to 245 K and characterised using 2D NMR. NOE connections were found from both hydride signals to the IMes resonances at δ 7.20, δ 6.88, δ 6.82, δ 2.29 and δ 2.18 and the bound sulfoxide resonance at δ 2.78 and δ 3.13. The relative integral intensities confirmed these were due to inequivalent CH_3 sites of the bound DMSO, rather than from two different ligated DMSO. Observation of nOe to both hydries confirms that the IMes and DMSO are *trans* and no nOe were observed between them. NOE from the hydride at δ −23.12 to the bound *ortho* signal of **A** at δ 8.83 confirmed the orientation of **A** *cis* to this hydride. ^{13}C NMR resonances were assigned using HMQC.

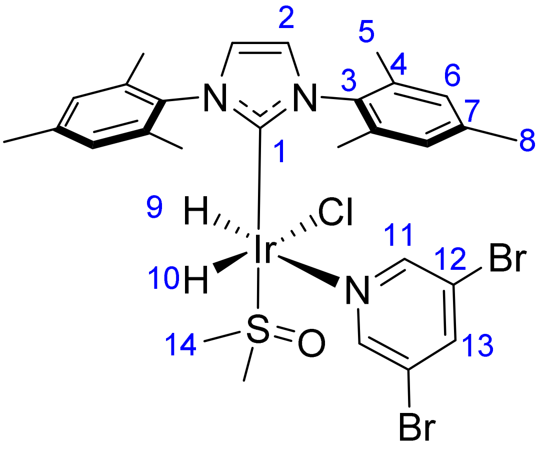

Figure S22: Structure of 8_B, its NMR resonances are given in Table S23.

Table S23: NMR resonances of 8_B. The resonance labels correspond to those shown in Figure S22.

	Methanol-d$_4$ at 245 K*	
**Resonance Number**	**^{1}H / ppm**	**^{13}C / ppm**
**1**		158.82
**2**	7.20	122.94
**3**		137.62
**4**		135.27, 135.50
**5**	2.16	17.33, 17.55
**6**	6.82, 6.88	128.35, 128.28
**7**		138.44
**8**	2.29	20.06
**9**	−23.55 (*d*, 7.5 Hz)	
**10**	−23.12(*d*, 7.5 Hz)	
**11**	8.83	-
**12**		120.17
**13**	8.25	140.01
**14**	2.78, 3.13	42.07, 54.91

*^{15}N signals in dichloromethane at 194.5 (IMes) and 257.8 (bound **B**)

S5.3: 2D NMR characterisation of 9_A

9_A was prepared by the reaction of **1** (10 mM), **A** (50 mM) and DPSO (25 mM) in methanol-*d*4 (0.6 mL) with H_2 (3 bar) over a few hours at room temperature, before being cooled to 245 K and characterised using 2D NMR. NOE connections were found from both hydride signals to the IMes resonances at δ 6.93, δ 6.84, δ 2.22 and δ 2.19 and the bound sulfoxide resonances at δ 7.39 and δ 7.44. Observation of nOe to both hydries confirms that the IMes and DPSO are *trans* and no nOe were observed between them. NOE from the hydride at δ −23.08 to the bound signals of **A** at δ 8.77 and δ 8.09 confirmed the orientation of **A** *cis* to this hydride. ^{13}C NMR resonances were assigned using HMQC.

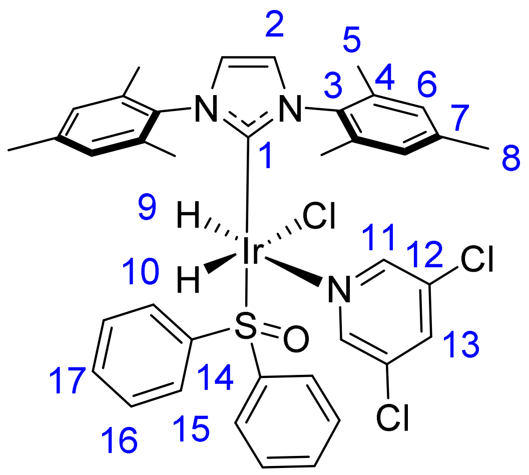

Figure S23: Structure of 9_A, its NMR resonances are given in Table S24.

Table S24: NMR resonances of 9_A. The resonance labels correspond to those shown in Figure S23.

	Methanol-d4 at 245 K	
**Resonance Number**	**^1H / ppm**	**^{13}C / ppm**
**1**		159.42
**2**	7.32	122.79
**3**		137.7
**4**		135.53, 135.80
**5**	2.22, 2.19	17.33/17.69
**6**	6.84/6.93	128.30/128.36
**7**		138.69
**8**	2.30	20.01
**9**	−22.28 (*d*, *J* = 7.3 Hz)	
**10**	−23.10 (*d*, *J* = 7.3 Hz	
**11**	8.77	-
**12**		-
**13**	8.09	-
**14**		145.20
**15**	7.39/7.44	125.92/126.04
**16**	7.2-7.3 (overlap)	130.39/130.82
**17**	7.2-7.3 (overlap)	-

S5.4: 2D NMR characterisation of 9_B

9_B was prepared by reaction of **1** (10 mM), **B** (50 mM) and DPSO (25 mM) in methanol-*d*4 (0.6 mL) with H_2 (3 bar) over a few hours at room temperature before being cooled to 245 K and characterised using 2D NMR. Characterisaton of **9_B** was challenging as when it was cooled to 245 K single crystals were formed in the NMR tube which affected the quality of the NMR data. Nonetheless, some 2D NMR data could be collected and this is shown in Table S25. NOE connections were found from both hydride signals to the IMes resonances at δ 6.96, δ 6.87, δ 2.24, and δ 2.32. NOE from the hydride at δ −23.08 to the bound signals of **B** at δ 8.89 and δ 8.22 confirmed the orientation of **A** *cis* to this hydride. ^{13}C NMR resonances were assigned using HMQC. Signals for the bound sulfoxide and the imidazole part of the IMes ligand could not be located due to peak overlap and poor quality NMR data likely caused by crystal formation. The structure of **9_B** was confirmed by X-ray crystallography (see section S5.5).

The crystal structures for **6_A**, **6_B** and **9_B** indicate iridium-nitrogen bond lengths for the dissociating ligands of 2.238(2), 2.148(5) and 2.2344(19) Å. Consequently, these bond lengths do not follow the trend in $\Delta H^{\neq}$ and suggests that other factors contribute to their values. Accordingly, substrate exchange could take an associative character, with other coordinating ligands such as methanol or even water playing a role. Any conclusive deductions will require a rigorous DFT study which is beyond the scope of this work.

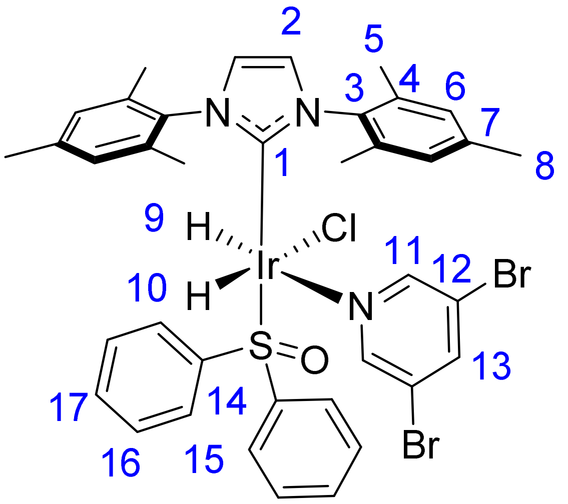

Figure S24: Structure of 9_B, its NMR resonances are given in Table S25.

Table S25: NMR resonances of 9_B. The resonance labels correspond to those shown in Figure S24.

Resonance Number	*Methanol-d4 at 245 K* ^{1}H / ppm	^{13}C / ppm
**1**		-
**2**	-	-
**3**		137.65
**4**		135.41, 135.94
**5**	2.24	17.41, 17.64
**6**	6.87, 6.96	128.37, 128.32
**7**		138.61
**8**	2.32	20.08
**9**	−22.25	
**10**	−23.08	
**11**	8.89	-
**12**		-
**13**	8.22	-
**14**		-
**15**	-	-
**16**	-	-
**17**	-	-

S5.5: X-Ray Crystallography of 9_B

9_B was prepared in methanol-*d*4 (0.6 mL) by reaction of [IrCl(η^2- η^2-COD)(IMes)] (**1**) (5 mM), DPSO (25 mM), and **B** (50 mM) with 3 bar H_2 and left at room temperature for 6 hours. At this point it was cooled to 278 K in a fridge and left for several weeks, at which point single crystals formed. A suitable crystal was selected and mounted on an Oxford-Diffraction SuperNova dual-source X-ray diffractometer equipped with copper and molybdenum sources and a HyPix-6000HE detector. Cooling to 110 K was achieved using an Oxford Instruments Cryojet. Using Olex2, the structure was solved with the SHELXT structure solution program using Intrinsic Phasing and refined with the SHELXL refinement package using Least Squares minimisation. Crystallography details are given in Table S26. The structure of **9_B** is shown in the main paper, Figure 3c.

Table S26: X-Ray Crystallography details for 9_B

Empirical formula	$C_{43}H_{42}Br_4ClIrN_4OS$
Formula weight/ Da	1210.15
Temperature/K	110.00(10)
Crystal system	triclinic
Space group	P-1
a/Å	8.5233(2)
b/Å	10.7222(2)
c/Å	23.8863(4)
α/°	98.3835(18)
β/°	97.7542(19)
γ/°	100.014(2)
Volume/Å^3	2097.72(8)
Z	2
ρ_{calc}g/cm^3	1.916
μ/mm^{-1}	11.984
F(000)	1172.0
Crystal size/mm^3	0.171 × 0.065 × 0.019
Radiation	Cu Kα (λ = 1.54184)
2Θ range for data collection/°	7.586 to 134.156
Index ranges	$-10 \le h \le 10$, $-10 \le k \le 12$, $-28 \le l \le 28$
Reflections collected	37630
Independent reflections	7482 [R_{int} = 0.0339, R_{sigma} = 0.0226]
Data/restraints/parameters	7482/0/511
Goodness-of-fit on F^2	1.039
Final R indexes [I>=2σ (I)]	R_1 = 0.0171, wR_2 = 0.0390
Final R indexes [all data]	R_1 = 0.0179, wR_2 = 0.0393
Largest diff. peak/hole / e Å^{-3}	0.55/-0.32
Ir-C_1 bond distance /Å	2.038
Ir-Cl bond distance /Å	2.473
Ir-N_{11} bond distance /Å	2.234
Ir-S bond distance /Å	2.254
Ir-H_9 bond distance /Å	1.445
Ir-H_{10} bond distance /Å	1.575

S5.6: PHIP time-courses for formation of 8_A

The formation of $\mathbf{8_A}$ was monitored at 245 K using PHIP by recording a series of single-scan ^{1}H NMR spectra at 245 K immediately after addition of *p*H_2 (3 bar) to a solution of pre-cooled **1** (5 mM), **A** (50 mM) and DMSO (25 mM) in methanol-*d*$_4$ (0.6 mL). Example spectra are shown in Figure S25.

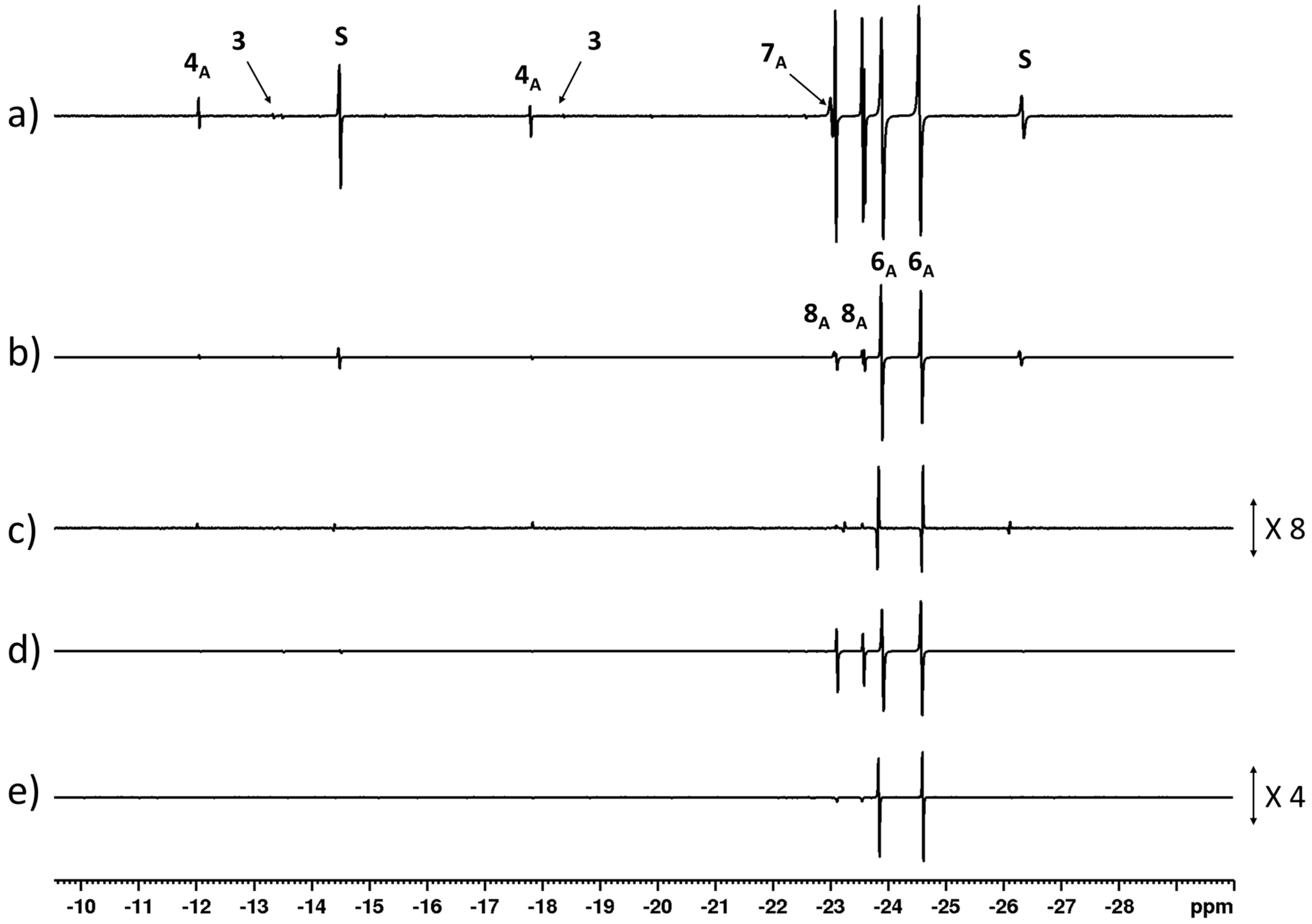

***Figure S25.** Series of single scan ^{1}H NMR spectra recorded with 45° pulses a) immediately after pH_2 (3 bar) to a solution of pre-cooled 1 (5 mM), A (50 mM) and DMSO (25 mM) in methanol-d$_4$ (0.6 mL) and shaken for 10 seconds at 6.5 mT. b) is recorded 10 seconds later and c) 2.5 minutes later. d) is recorded after shaking with fresh pH2 and is recorded 10 minutes after a). e) is recorded 1.5 minutes after d). The species labelled S is likely an analogue of 5 where L is replaced with DMSO.*

S6: Ligand loss rates in 6, 8 and 9

The dissociation of **A** or **B** from either **6**, **8** or **9** was measured using exchange spectroscopy (EXSY). This involved the selective excitation the *ortho* protons of bound **A** or **B** *trans* to hydride, followed by a variable delay time, before a ^{1}H NMR spectrum is recorded. Peaks are observed for excited metal complex, and as the delay time is increased a signal for free ligand that was previously bound to the metal centre becomes visible. The proportion of bound and free ligand as a function of delay time is measured and fitted to a kinetic model to extract a kinetic dissociation rate. The rate is found by minimising the difference between experimentally determined bound and free ratios, and those predicted by a kinetic two site exchange model. Errors were calculated using the Jack Knife approach. This involves sequentiually removing one data point, calculating the rate constant, and then taking an error of all of the rate constants. The enthalpy ($^{\pm}$) and entropy (S $^{\pm}$) were determined by recording the dissociation rate, k, at different temperatures, T, and fitting the data to a linearized Eyring equation (equation 1) where $_B$ is Boltzmann's constant, h is Planck's constant, and is the ideal gas constant. Accordingly, a plot of $\ln\left(\frac{2k}{T}\right)$ against $\frac{1}{T}$ yields a straight line with a gradient of $-\frac{\Delta H^{\pm}}{R}$ and a y axis intercept of $\ln\left(\frac{k_B}{h}\right) + \frac{\Delta S^{\pm}}{R}$, allowing calculation of the entropy and enthalpy. As the experiments measure the forward rate constant crossing the activation barrier, a factor of 2 in included to account for the equal probability of the symmetric transition state reacting onwards to give an exchange product, or going backwards to reform the starting materials.

$$\ln\left(\frac{}{}\right) = \ln\left(\frac{_B}{h}\right) + \frac{S^{\pm}}{} - \frac{^{\pm}}{} \qquad (1)$$

Samples used to determine these exchange rates contained **1** (5 mM) and **A** or **B** (45 mM) with H_2 (3 bar) in dichloromethane-*d*$_2$ (for exchange rates of **6**) and **1** (5 mM) and either **1** (5 mM), **A** or **B** (15 mM) and sulfoxide (100 mM) with H_2 (3 bar) in methanol-*d*$_4$ or **1** (5 mM), **A** or **B** (80 mM) and sulfoxide (100 mM) with H_2 (3 bar) in dichloromethane-*d*$_2$ (for exchange rates of **8** or **9**). Exchange rates for **9$_A$** could not be determined due to the inability to selectively excite **A** within **9$_A$** due to peak overlap (with the DPSO resonances).

Table S27. Rate of substrate dissocation determined from EXSY data with corresponding enthalpy and entropy changes of ligand dissociation.

Temp /K	**6$_A$**	**6$_B$**	**8$_A$**		**8$_B$**		**9$_B$**
	DCM-d_2	DCM-d_2	Methanol-d_4	DCM-d_2	Methanol-d_4	DCM-d_2	DCM-d_2
303	-	-	12.52 ± 0.17	33.93 ± 0.53	8.18 ± 0.28	-	
298	-	-	-	-	4.38 ± 0.18	17.1 ± 0.27	-
288	-	-	6.74 ± 0.02	5.44 ± 0.36	1.44 ± 0.04	4.72 ± 0.08	1.31 ± 0.08
283	-	-	0.98 ± 0.01	-	-	-	-
278	-	-	-	1.53 ± 0.01	0.44 ± 0.01	1.31 ± 0.01	0.37 ± 0.01
273	-	-	0.29 ± 0.01	-	-	-	-
268	5.62 ± 0.30	9.62 ± 0.35	-	0.34 ± 0.01	0.098 ± 0.001	0.28 ± 0.01	0.095 ± 0.01
263	2.54 ± 0.13	-	0.064 ±	-	-	-	-
258	1.06 ± 0.05	2.01 ± 0.02	0.002	0.073 ± 0.002	0.020 ± 0.001	0.063 ± 0.001	0.021 ± 0.001
248	0.084 ± 0.004	0.41 ± 0.01	-	0.014 ± 0.001	-	0.015 ± 0.001	0.0037 ± 0.001
238	0.035 ± 0.002	0.075 ± 0.001	-	-	-	-	
233	-	0.029 ± 0.001	-	-	-	-	
			-				
ΔH$^{\neq}$ /kJ mol^{-1}	88.1 ± 2.0	83.3 ± 2.0	84.2 ± 1.0	85.7 ± 1.0	83.5 ± 2.0	85.3 ± 1.5	83.1 ± 1.0
ΔS$^{\neq}$ /J K^{-1} mol^{-1}	105 ± 8	93 ± 8	59.5 ± 4.0	72.6 ± 3.0	53.8 ± 7.0	70.4 ± 5.8	52.0 ± 3.0

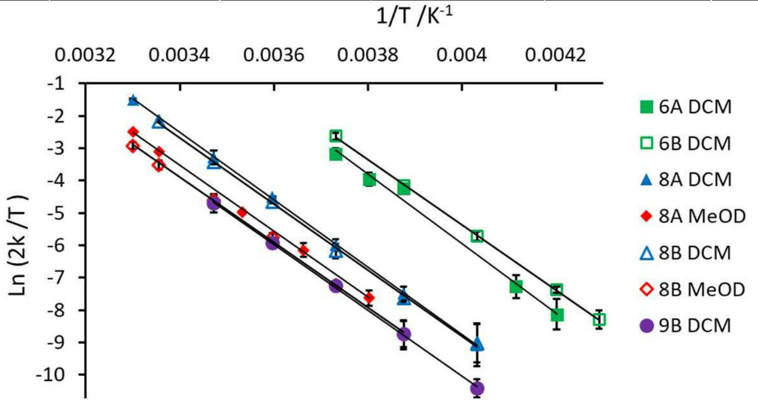

Figure S26. Linearised Eyring plot linking the rates of substrate dissociation as determined by EXSY at the specified temperature. The gradients and intercepts are used to calculate the entropy and enthalpy of ligand dissociation.

S7: SABRE Hyperpolarisation of A and B in different solvents

Samples containing [IrCl(η²-η²-COD)(IMes) (**1**) (5 mM) and the substrate 3,5-dichloropyridine (**A**) or 3,5-dibromoopyridine (**B**) (50 mM) with DMSO (25 mM) were dissolved in either dioxane, tetrahydrofuran, or tetrahydrofuran-d_8 (0.6 mL) and exposed to 3-bar H_2 gas for several hours to form **8**. After this point, the H_2 atmosphere was replaced with $p H_2$ and a series of hyperpolarisation measurements were performed by shaking the sample for 10 seconds in the stray field of a 9.4 T spectrometer (*ca* 6.5 mT) (for ^{1}H), a mu metal shield at 1 µT (for ^{13}C) or a mu metal shield at 6 µT (for ^{15}N). NMR spectral acquisition commenced immediately after the sample was dropped into the spectrometer, and the hyperpolarisation process was repeated multiple times for each sample by replacing the spent $p H_2$ with fresh $p H_2$ before re-shaking the solution as described.

^{1}H, ^{13}C, and ^{15}N NMR signal enhancements for **A** and **B** are given in Tables S28, S29 and S30 respectively. ^{1}H T_1 relaxation times are also given in Table S28. A graphical representation is shown in Figure S27.

Table S28: ^{1}H NMR signal enhancements and T_1 relaxation times for A and B (50 mM) recorded by shaking a sample of [IrCl(η²-η²-COD)(IMes)] (5 mM) and substrate with DMSO (5 eq) in the indicated solvent with 3-bar pH_2 for 10 seconds in the fringe field of a 9.4 T magnet (ca 6.5 mT).

Solvent	Site	**A** 50 mM				**B** 50 mM			
		Ortho Bound	*Ortho* Free	*Para* Bound	*Para* Free	*Ortho* Bound	*Ortho* Free	*Para* Bound	*Para* Free
Dioxane	^{1}H E/ fold	187 ± 17	563 ± 41	361 ± 24	66 ± 5	-	583 ± 243	525 ± 41	-
THF	^{1}H E/ fold	622 ± 42	1339 ± 108	1484 ± 349	188 ± 102	2512 ± 23	1855 ± 41	994 ± 40	358 ± 37
THF-d_8	^{1}H E/ fold	1472 ± 163	1740 ± 294	1081 ± 314	674 ± 54	1892 ± 444	2135 ± 37	1437 ± 287	2131 ± 1259

Table S29: ^{13}C NMR signal enhancements for A and B (50 mM) recorded by shaking a sample of [IrCl(η²-η²-COD)(IMes)] (5 mM) and substrate with DMSO (5 eq) in the indicated solvent with 3-bar pH_2 for 10 seconds at 1 µT.

Solvent	**A** 50 mM	**B** 50 mM
Dioxane	carbene: 0<br>o free: 25 ± 1<br>o bound: 30 ± 7<br>p free: 155 ± 7<br>p bound: 0<br>m free: 147 ± 15<br>m bound: 0	carbene: 7 ± 1<br>o free: 10 ± 1<br>o bound: 14 ± 1<br>p free: 4 ± 2<br>p bound: 5 ± 1<br>m free: 197 ± 16<br>m bound: 6 ± 1
THF	carbene: 42 ± 3<br>o free: 297 ± 5<br>o bound: 326 ± 18<br>p free: 90 ± 3<br>p bound: 113 ± 1<br>m free: 276 ± 28<br>m bound: 0	carbene: 59 ± 1<br>o free: 384 ± 49<br>o bound: 416 ± 51<br>p free: 131 ± 20<br>p bound: 168 ± 19<br>m free: 684 ± 17<br>m bound: 0
THF-d_8	carbene: 62 ± 2<br>o free: 466 ± 47<br>o bound: 506 ± 56<br>p free: 120 ± 26<br>p bound: 169 ± 23<br>m free: 550 ± 70<br>m bound: 0	carbene: 49 ± 25<br>o free: 284 ± 142<br>o bound: 298 ± 150<br>p free: 70 ± 36<br>p bound: 150 ± 75<br>m free: 516 ± 258<br>m bound: 29 ± 15

***Table S30:** ^{15}N NMR signal enhancements for A and B (50 mM) recorded by shaking a sample of [IrCl(η^2-η^2-COD)(IMes)] (5 mM) and substrate with DMSO (5 eq) in the indicated solvent with 3-bar pH_2 for 10 seconds at 6 μT*

Solvent	**A** 50 mM	**B** 50 mM
Dioxane	Free: 1588 ± 136	Free: 3670 ± 70
THF	Free: 7486 ± 70<br>Bound: 463 ± 32	Free: 7342 ± 288<br>Bound: 521 ± 140
THF-d_8	Free: 7928 ± 299<br>Bound: 603 ± 37	Free: 7851 ± 195<br>Bound: 620 ± 24

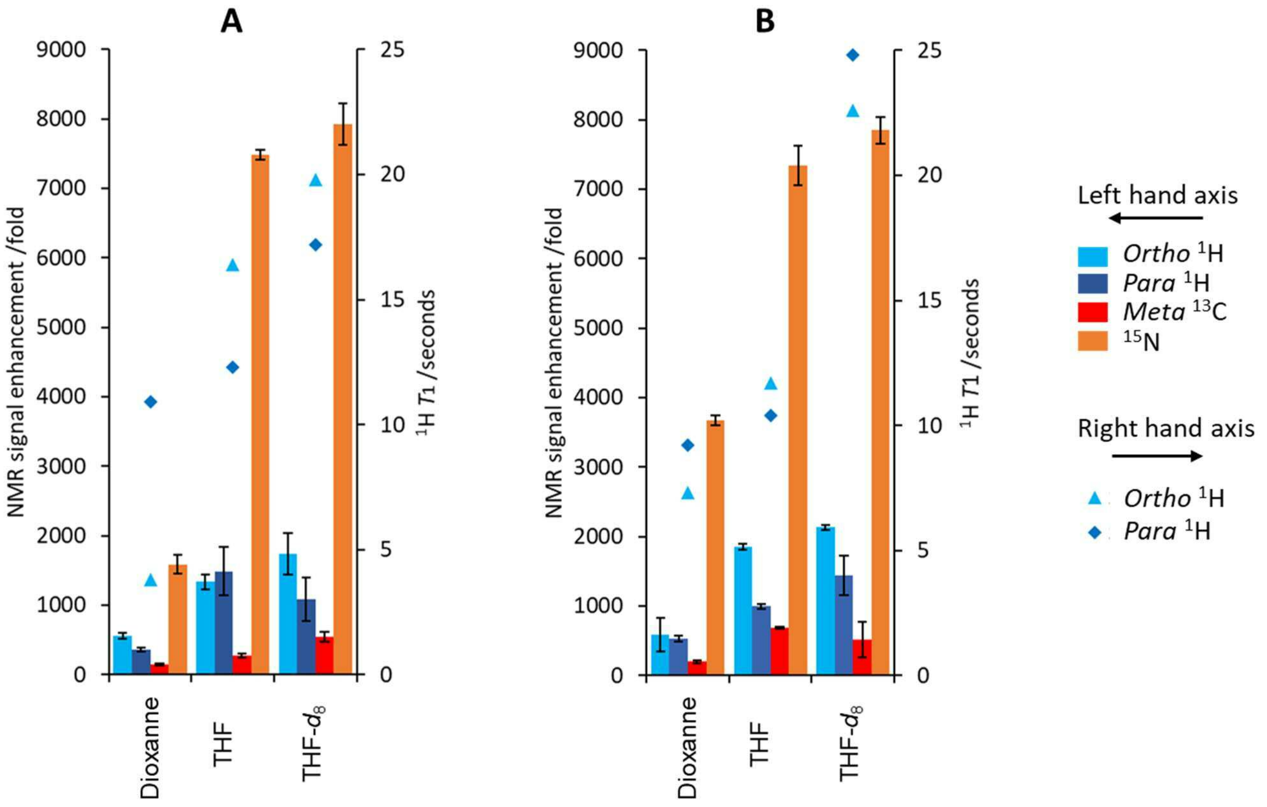

***Figure S27:** ^1H, ^{13}C, and ^{15}N NMR signal enhancements for A (left) and B (right) (50 mM) recorded by shaking a sample of [IrCl(η^2-η^2-COD)(IMes)] (5 mM) and substrate with DMSO (5 eq) in the indicated solvent with 3-bar pH_2 for 10 seconds at either 6.5 mT (^1H) 1 μT (^{13}C) or 6 μT (^{15}N).*

S8: Methylation of 3,5-dichloropyridine

After addition of $CF_3SO_2OCH_3$ with **A** (in the presence of $\mathbf{8_A}$) in THF-d_8 as detailed in the main paper, crystals were observed to form over time. X-ray crystallography revealed them to be the methylated product, with the demethylated reagent also present in the unit cell (Figure S28, details shown in Table S31). A suitable crystal was selected and mounted on an Oxford-Diffraction SuperNova dual-source X-ray diffractometer equipped with copper and molybdenum sources and a HyPix-6000HE detector. Cooling to 110 K was achieved using an Oxford Instruments Cryojet. Using Olex2, the structure was solved with the SHELXT structure solution program using Intrinsic Phasing and refined with the SHELXL refinement package using Least Squares minimisation. Crystals were found to be a Non-merohedral twin modelled with two components in the ratio 0.5068:0.4932(16).

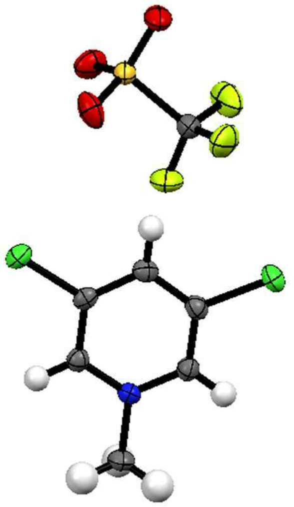

Figure S28: X-Ray Crystallography structure with thermal ellipsoids shown at 50% probability. Grey corresponds to carbon, blue to nitrogen, green to chlorine, white to hydrogen, yellow to sulfur, red to oxygen and light green to fluorine.

Table S31: X-Ray Crystallography details for the methylated product.

Empirical formula	$C_7H_6NO_3F_3SCl_2$
Formula weight/ Da	312.09
Temperature/K	110.05(10)
Crystal system	monoclinic
Space group	$P2_1/c$
a/Å	6.6816(2)
b/Å	12.5366(6)
c/Å	13.5582(5)
α/°	90
β/°	94.793(3)
γ/°	90
Volume/Å^3	1131.73(7)
Z	4
ρ_{calc}g/cm^3	1.832
μ/mm^{-1}	7.303
F(000)	624.0
Crystal size/mm^3	0.264 × 0.038 × 0.035
Radiation	Cu Kα (λ = 1.54184)
2Θ range for data collection/°	9.624 to 134.05
Index ranges	−7 ≤ h ≤ 7, −14 ≤ k ≤ 14, −16 ≤ l ≤ 16
Reflections collected	3310
Independent reflections	3310 [R_{int} = ?, R_{sigma} = 0.0148]
Data/restraints/parameters	3310/0/156
Goodness-of-fit on F^2	1.065
Final R indexes [I>=2σ (I)]	R_1 = 0.0385, wR_2 = 0.1141
Final R indexes [all data]	R_1 = 0.0426, wR_2 = 0.1176
Largest diff. peak/hole / e Å^{-3}	0.34/-0.41

Mass spectrometry on the crystals also confirmed the presence of **A-CH_3** (Figure S29). Calculated $[C_6H_6Cl_2N]^+$ m/z 161.9877, found $[C_6H_6Cl_2N]^+$ m/z 161.9875.

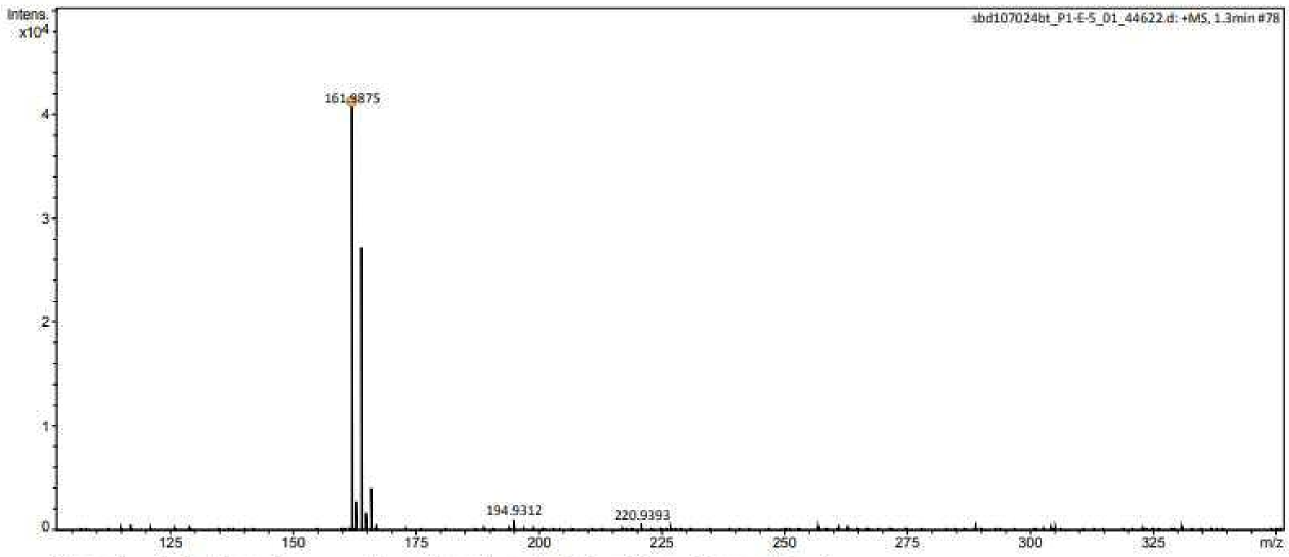

Meas. m/z	#	Ion Formula	m/z	err [ppm]	err [mDa]	mSigma	Mean err [ppm]
161.9875	1	C6H6Cl2N	161.9872	-1.7	-0.3	11.8	-2.2

Figure S29: ESI Mass spectrum of A-CH_3
